# Understanding Fenofibrate Release from Bare and Modified Mesoporous Silica Nanoparticles

**DOI:** 10.3390/pharmaceutics15061624

**Published:** 2023-05-30

**Authors:** Giorgia Figari, José L. M. Gonçalves, Hermínio P. Diogo, Madalena Dionísio, José Paulo Farinha, María Teresa Viciosa

**Affiliations:** 1 Centro de Química Estrutural, Complexo I, Instituto Superior Técnico, University of Lisbon, Avenida Rovisco Pais, 1049-001 Lisbon, Portugal; 2 LAQV-REQUIMTE, Department of Chemistry, NOVA School of Science and Technology, Universidade Nova de Lisboa, 2829-516 Caparica, Portugal

**Keywords:** mesoporous silica nanoparticles, confined water, fenofibrate, amorphous state, drug release

## Abstract

To investigate the impact of the surface functionalization of mesoporous silica nanoparticle (MSN) carriers in the physical state, molecular mobility and the release of Fenofibrate (FNB) MSNs with ordered cylindrical pores were prepared. The surface of the MSNs was modified with either (3-aminopropyl) triethoxysilane (APTES) or trimethoxy(phenyl)silane (TMPS), and the density of the grafted functional groups was quantified via ^1^H-NMR. The incorporation in the ~3 nm pores of the MSNs promoted FNB amorphization, as evidenced via FTIR, DSC and dielectric analysis, showing no tendency to undergo recrystallization in opposition to the neat drug. Moreover, the onset of the glass transition was slightly shifted to lower temperatures when the drug was loaded in unmodified MSNs, and MSNs modified with APTES composite, while it increased in the case of TMPS-modified MSNs. Dielectric studies have confirmed these changes and allowed researchers to disclose the broad glass transition in multiple relaxations associated with different FNB populations. Moreover, DRS showed relaxation processes in dehydrated composites associated with surface-anchored FNB molecules whose mobility showed a correlation with the observed drug release profiles.

## 1. Introduction

Aiming to improve the therapeutic activity of already-established drugs, yet limited in their activity, the pharmaceutical industry has redirected a good part of its research to the development of new formulations, saving the cost associated with the discovery of new ingredients.

Examples of this strategy are formulations that explore the possibility of using the drug in metastable states or in the solid amorphous form, whose excess free energy in relation to the more stable crystalline counterpart may result in improved solubility and/or dissolution rate [1]. However, because the solid amorphous form is out of thermodynamic equilibrium, it can evolve to the more stable crystalline state or, in the case of crystalline metastable states, to another polymorph. Therefore, to benefit from amorphization, efforts have been made to reduce the driving force to recrystallization, whether it takes place during manipulation and storage or in the dissolution steps. This can be achieved via incorporation in nanoporous matrices [2,3,4]. The amorphization of the guest occurs via a spatial limitation, i.e., the pore size is small enough in relation to the critical nucleation size, preventing the rearrangement of the disordered molecules into long-range ordered structures, or inducing the formation of a different crystalline network (polymorphism) [5]. The reduced cooperative mobility of the loaded drug may influence the tendency to organize in a definite manner, impairing the formation of a crystal lattice structure [6]. In fact, low molecular mobility, which is a measure of the rate of cooperative rearrangements in the amorphous state, and high configurational entropy, the entropy difference between the disordered and crystalline states, were identified by Zhou and co-workers [7] as key factors governing the avoidance of crystallization. This study was carried out on several drugs including fenofibrate (FNB), the target compound tested in the present work, a pro-drug which undergoes hydrolysis to active fenofibric acid, which has been prescribed since the 1980s to treat hypercholesterolemia [8,9].

**Scheme 1:**
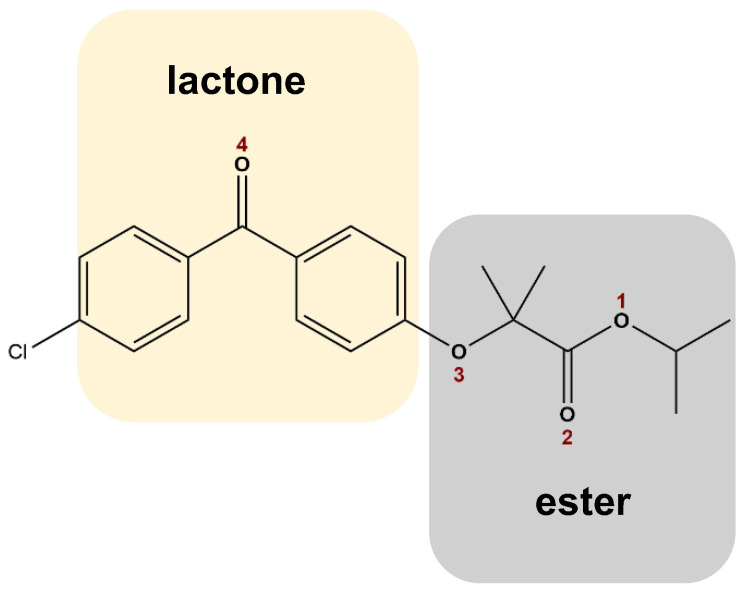
Fenofibrate (propan-2-yl 2-(4-[(4-chlorophenyl)carbonyl]phenoxy)-2-methylpropanoate) chemical structure with oxygen atoms numbered.

In accordance with the Biopharmaceutical Classification System, FNB (Scheme 1) belongs to class II due to its high permeability through lipid membranes (>90%) and its poor aqueous solubility (0.8 μg mL^−1^) [10], a drawback that could reduce the drug’s bioavailability. Therefore, many recent formulations have been developed [9,11,12]. Among these, there have been several attempts reported in the literature to explore FNB in the amorphous state by incorporating it in silica matrices [13,14]. These drug carriers present significant low cytotoxicity [15,16,17] and a high surface area, allowing for a high dispersion of the loaded compound. However, the crystallization tendency has not been totally suppressed in silica-based FNB formulations [18,19]. Despite this, silica-based FNB formulations have shown improved solubility and increased bioavailability, as well as high potential in clinical studies [20,21].

Motivated by such promising results using mesoporous silica as a carrier for FNB, it is intended to deeply understand how the matrix characteristics in terms of pore geometry and surface treatment influence the guest’s physical state, molecular mobility and, ultimately, its release under physiological conditions, trying to provide a rational basis for the future design of drug delivery systems. Neat FNB can be taken as a model compound due to its glass-forming ability, allowing it to test its molecular mobility in amorphous form [22,23], but with a tendency for crystallization when kept close to room temperature.

Therefore, spherical mesoporous silica nanoparticles (MSNs) with cylindrical pore diameters ~3 nm were prepared. The pore wall was functionalized to evaluate the impact of the matrix surface on the drug release profile compared with the bare nanoparticles. Aiming for this, the bare MSNs were chemically modified replacing some silanol groups with, respectively, hydrophilic (3-aminopropyl) triethoxysilane (APTES) or hydrophobic trimethoxy(phenyl)silane (TMPS); the functionalized nanoparticles will be hereafter designated as MSN_APTES and MSN_TMPS. As silanol density impacts water diffusivity and surface wettability [24], the water dynamical behavior was probed via dielectric relaxation spectroscopy (DRS) as a mean to evaluate the influence of APTES or TMPS grafting.

The bare and functionalized MSNs were loaded with FNB and the obtained composites were characterized by a set of experimental techniques. FTIR and DSC were used to access the physical state of the loaded FNB, and its mobility was probed via DRS, in both hydrated and dehydrated states, allowing for the correlation of the guest dynamics with the observed release profiles. Since the pore size is of the order of a few nanometers, the work provides fundamental information regarding finite size effects from which a length scale for crystallization and glass transition can be inferred.

## 2. Materials and Methods

### 2.1. Materials

Fenofibrate, IUPAC name propan-2-yl 2-[4-(4-chlorobenzoyl)phenoxy]-2-methylpropanoate (CAS number 49562-28-9), was purchased from Sigma-Aldrich, St. Louis, MO, USA (purity ≥ 99%) and was used without further purification. The empirical formula is C_20_H_21_ClO_4_, and the molecular weight is 360.83 g mol^−1^. Absolute ethanol (EtOH, >99.9% Scharlau), tetraethoxysilane (TEOS, ≥99%, Sigma-Aldrich GHEMIE GmbH, Steinheim, Germany), cetyltrimethylammonium bromide (CTAB, ≥99%, Sigma, St. Louis, MO, USA) and sodium hydroxide solution (NaOH 1.4 M) were used without any purification in the synthesis of the mesoporous silica nanoparticles. For the removal of the surfactant, a solution of 0.5 M hydrochloric acid (HCl, 37% Panreac AppliChem, Darmstad, Germany) in absolute ethanol was used. The deionized water was produced from a Millipore system Milli-Q ≥18 MΩcm (with a Millipak membrane filter 0.22 µm). Regarding nanoparticle functionalization, they were surface modified with trimethoxy(phenyl)silane (97% TMPS, Sigma-Aldrich) and (3-Aminopropyl) triethoxysilane (98% APTES, Sigma-Aldrich) without any treatment in dried toluene, which was distilled over calcium hydride before use. For the NMR analysis samples, we used 1,3,5-trioxane (≥99.0%, Fluka, Seelze, Germany), deuterium oxide (D_2_O, 99.9% atom, CIL) and dimethyl sulfoxide D6 (DMSO, 99.9%, CIL). Disodium hydrogen phosphate (Na_2_HPO_4_, 99%, Riedel-de-Haën, Seelze, Germany) and sodium dihydrogen phosphate monohydrate (NaH_2_PO_4_.H_2_O, 98%, Panreac, Castellar del Vallès, Spain) were used to prepare the phosphate-buffered solution (PBS, pH 8) for the release studies.

### 2.2. Equipment

Centrifugation. An Avanti J-301 (Beckman Coulter, Brea, CA, USA) was used for cleaning the MSNs after template removal. For the centrifugations, 50 mL centrifuge propylene tubes were used. A Sigma 2k15 (B. Braun, Laguna Hills, CA, USA) centrifuge, rotor 12,141, was used for washing the functionalized MSNs at 1100 min^−1^. Disposable 10 mL polypropylene tubes were used for the centrifugations. A Hitachi Himac CT 15RE centrifuge was utilized with 2 mL Eppendorf for washing bare and functionalized nanoparticles after drug loading at 15,000 rpm.

pH measurements. The pH of the phosphate-buffered solution for the release studies was measured using a pHenomenal^®^, 1000 L bench pH (mV°C)^−1^ meter pH.

Transmission electron microscopy (TEM). TEM images were obtained using a Hitachi transmission electron microscope (Hitachi High-Technologies, Tokyo, Japan), model H-8100, with a LaB6 filament (Hitachi) and an accelerator voltage of 200 kV. A camera KeenView (Soft Imaging System, Münster, Germany) is incorporated into this equipment, which via iTEM software allows one to acquire TEM images. MSNs dispersed in ethanol were prepared and dried on a copper grid coated with carbon. The size/dimension, polydispersity and morphology of the particles were estimated using ImageJ 1.50i Fiji software (Madison, WI, USA).

Nuclear magnetic resonance (^1^H NMR). The spectra were recorded using an AMX-400 instrument (Bruker, MA, USA) at 400 MHz. For this purpose, two solutions of NaOH and 1,3,5-trioxane (internal standard, IS) in deuterated water (D_2_O) were prepared. In an NMR tube, 5 mg of the sample, 400 μL of a solution of NaOH and 100 μL of a solution of 1,3,5-trioxane in D_2_O were mixed and sonicated.

Fourier transform infrared spectroscopy (FTIR). The FTIR spectra of pure MSNs, native crystalline FNB and FNB loaded in nanoparticles, after solvent evaporation, were collected using a Bruker (model: Alpha) over the range 4000–400 cm^−1^ at room temperature in the form of KBr pellets.

Differential scanning calorimetry (DSC). The thermal features were examined using a differential scanning calorimeter DSC Q2000 from TA Instruments Inc. Guyancourt, France (Tzero DSC technology), operating in the Heat Flow T4P option. Enthalpy (cell constant) and temperature calibration were based on the melting peak of indium standard (T_m_ = 156.60 °C). Approximately 5 mg of each sample was introduced in an aluminum hermetic pan with a Tzero hermetic lid and a pinhole to facilitate the exit of water. Thermograms of all of the samples were obtained over a range of −90 °C to 120 °C at a heating rate of 10 °C min^−1^ under a nitrogen flow of 50 mL min^−1^ and they were analyzed during heating. Then, the analysis of data was carried out using the software Universal Analysis 2000 from Thermal Analysis. The melting point (T_m_) was determined as the minimum of the endothermic peak, whereas the glass transition temperature (T_g_) was determined at the onset of the glass transition.

Dielectric relaxation spectroscopy (DRS). Dielectric measurements were carried out using the ALPHA-N impedance analyzer from Novocontrol Technologies GmbH, covering a frequency range from 0.1 Hz to 1 MHz. The sample powder was placed between two gold-plated electrodes of a parallel plate capacitor, BDS 1200, with two 50 μm thick silica spacers, and the sample cell was mounted on a BDS 1100 cryostat and exposed to a heated gas stream being evaporated from a liquid nitrogen dewar. The temperature was controlled using the Quatro Cryosystem and performed at 0.5 °C.

To analyze the isothermal dielectric data, the Havriliak–Negami (*HN*) model function [25,26] was fitted to both imaginary and real components of the electrical modulus, $M_{HN}^{*}\left(\omega \right)$. To account for the multimodal profile, a sum of *HN* functions was considered:

(1) $$M_{HN}^{*}\left(\omega \right)=M_{\infty }+\sum_{j}\frac{∆M}{{\left(1+{\left(-i{\left(\omega \tau_{HN}\right)}^{-1}\right)}^{\alpha_{HN}}\right)}^{\beta_{HN}}}$$

where *j* is the index over which the individual processes are summed, Δ*M* = *M*_0_ − *M*_∞_, τ_HN_ is the characteristic *HN* relaxation time and *α_HN_* and *β_HN_* are shape parameters (0 < *α_HN_* < 1 and 0 < *α_HN_ β_HN_* ≤ 1) describing, respectively, the symmetric and asymmetric broadening of the complex dielectric function [27].

From the estimated values of *τ_HN_* and the shape parameters, a model-independent relaxation time was determined from [25]:

(2) $$\tau_{max}=\tau_{HN}{\left[sin\left(\frac{\alpha_{HN}\pi }{2+2\beta_{HN}}\right)\right]}^{-\frac{1}{\alpha_{HN}}}{\left[sin\left(\frac{\alpha_{HN}\beta_{HN}\pi }{2+2\beta_{HN}}\right)\right]}^{-\frac{1}{\alpha_{HN}}}$$

The temperature dependence of the relaxation times could be described using the Arrenhius equation in the case of thermally activated processes (linear T-dependence) or the well-known Vogel/Fulcher/Tammann/Hesse equation [28,29,30] when curved, respectively; see Equations (3) and (4).

(3) $$\tau =\tau_{0}exp\left(\frac{E_{a}}{RT}\right)$$

where *τ*_0_ is the relaxation time at infinite *T*, *R* is the ideal gas constant and *E_a_* is the activation energy, and

(4) $$\tau \left(T\right)=\tau_{0}exp\left(\frac{B}{T-T_{0}}\right)$$

where *τ*_0_ and *B* are parameters and *T*_0_ is the so-called Vogel temperature (found around 50 degrees below *T*_g_).

### 2.3. Methods

Synthesis and characterization of the MSNs. Mesoporous silica nanoparticles (MSNs) were synthesized following a method reported in [31,32]. Briefly, 1.75 mL NaOH 1.4 M and 240 mL H_2_O milli-Q were mixed in a polypropylene flask with a flat bottom, which was transferred to an oil bath and heated to 32 °C. The reaction was performed with homogeneous magnetic stirring to obtain a stable mixture and avoid a larger distribution of diameters. Afterward, 500 mg of cetyltrimethylammonium bromide (CTAB) was added to the solution and maintained under stirring for 30 min. Then, 2.5 mL of tetraethyl orthosilicate (TEOS) was added, drop by drop, and the solution was kept under continuous stirring for 3 h at 35 °C. The particles were recovered via centrifugation and the solid was washed with a mixture of ethanol and water (50% *v*/*v*). The solid (MSNs-CTAB) was dried at 50 °C overnight and the latter was placed under vacuum. Then, 0.5 M HCl in ethanol (25 mL per 500 mg of MSNs) was added to solubilize the CTAB and the solution was sonicated for 15 min and left stirring overnight at 50 °C. After 24 h, the particles were washed 3 times. First, with a solution of ethanol and water (50% *v*/*v*), and then twice with absolute ethanol. Finally, the nanoparticles were dried overnight in a ventilated oven at 50 °C.

Modification of the MSNs pores. The modification of the MSNs’ surface with (3-aminopropyl)triethoxysilane (APTES) was performed by suspending 0.166 g of the MSNs in anhydrous toluene (10 mL) under an argon atmosphere into a 25 mL round bottom flask. This flask was sonicated under argon atmosphere for 15 min. Subsequently, APTES (0.10 mL) was added dropwise, and the resulting mixture was maintained at 130 °C under reflux in an argon atmosphere for 24 h. A solid product (MSN_APTES) was obtained via centrifugation (1100 min^−1^ for 10 min) and washed three times with absolute ethanol, discarding the supernatant each time. The particles were dried in a ventilated oven for 24 h at 50 °C.

The surface of the MSNs was also covalently modified with trimethoxy(phenyl)silane (TMPS). For this purpose, 0.169 g of nanoparticles was dispersed in 15 mL of dry toluene. Then, 50 μL of TMPS was added to the mixture and it was maintained at 125 °C under reflux in an argon atmosphere for 24 h. The remaining steps were like those carried out with MSN_APTES. Solid MSN_TMPS was recovered.

Loading MSNs with FNB. To remove any impurities, or residual solvents including water, the mesoporous nanoparticles were placed in a glass cell under vacuum (10^−4^ bar) and heated up to 150 °C via the immersion of the cell in an oil bath, for 8 h. After this period, the cell was allowed to cool down to room temperature. FNB dissolved in 2 mL of acetone was injected in the cell container hosting the outgassed matrix and then stirred for 3 h to increase the uptake of the drug. Finally, the solvent was allowed to evaporate for one day under gentle stirring at room temperature, and after which a dry powder was obtained (FNB@MSNs). Table 1 presents the drug loading conditions used for all of the silica nanocarriers.

The theoretical maximum of the loading was estimated as in [33]:

(5) $$\text{maximum loading}=\left[\frac{V_{p}*\rho_{FNB}}{1g+V_{p}*\rho_{FNB}}\right]*100\%$$

where *ρ_FNB_* is the density of the fenofibrate (1.177 g cm^−3^ taken from reference [34] for form I of crystalline FNB) and *V_p_* is the pore volume of the silica. The latter (*V_p_* = 0.68 cm^3^ g^−1^) was estimated from BET data for similar mesoporous silica nanoparticles, as described by some of us in reference [32]. The maximum theoretical load calculated from Equation (5) was approximately 44 wt%. However, it must be noted that the maximum loading may have been overestimated, because the density of the crystalline FNB, used to determine it, was higher than that of the amorphous form [34].

In the functionalized MSNs, the pore diameter can slightly decrease due to the anchoring of the modifying agent. This effect has been reported for mesoporous silica modified with methyltrimethoxysilane, which reduced the pore diameter by 0.2 nm [35]. Having this in mind, FNB was loaded significantly below the theoretical maximum predicted by Equation (5) to minimize the deposition outside the pores (both FNB mass and % occupied volume are included in Table 1).

Monitoring drug release. Measurements were performed at room temperature using a UV-660 UV-Vis Spectrophotometer (JASCO International, Tokyo, Japan) with a double monochromator and a photomultiplier detector. The assays were carried out at room temperature in a quartz cuvette with 1 cm × 1 cm dimension using a polypropylene dialysis device with a cellulose membrane (Slide-A-Lyzer Mini Dialysis Devices, 10 K MWCO, 0.5 mL) inserted in its top (a schematic representation of this setup is illustrated in the SI of ref. [36]). The solution medium for drug release was composed of phosphate buffer at pH 7.4 and ethanol in the w/w proportion of 90:10; a calibration curve at λ = 289 nm was constructed by dissolving known FNB amounts in the same solution medium with the following linear regression parameters: *A* = (15.2 ± 0.5) × 10^3^ × *C* + (0.005 ± 0.003), where the slope represents ε (molar absorptivity coefficient) and C is the concentration. Ethanol was chosen due to the higher solubility of FNB in this medium [37]. Briefly, 200 µL of PBS/EtOH was added to around 1.1 mg of loaded nanoparticles and placed in the top chamber. The bottom chamber was filled with 2.8 mL of the buffered solution media and kept under continuous stirring at 400 rpm. Drug release was monitored at room temperature (~25 °C) by collecting successive UV-Vis spectra (200 to 700 nm) at an acquisition rate of 400 nm min^−1^. During the first 4 h, spectra were taken every 60 s and then every 600 s until completion at 8 h.

## 3. Results

### 3.1. Structural Characterization of MSNs

**Figure 1:**
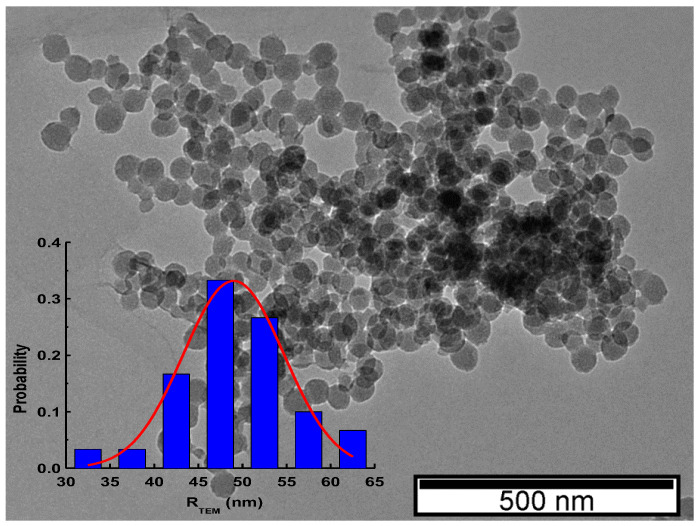
Transmission electron microscope image illustrating the regular shape and size of the mesoporous silica nanoparticles. Inset: probability distribution of nanoparticles’ radius obtained from a sample of 30 nanoparticles; the red line corresponds to the Gaussian fitting function of μ = 49 and σ^2^ = 32.6, corresponding to a particle radius R = 49 ± 6 nm.

Mesoporous silica nanoparticles were observed via TEM (representative image in Figure 1) which confirmed the spherical shape of the nanoparticles and allowed for the determination of their size. The analysis of diameter using ImageJ software provided a value of 49.0 nm (inset of Figure 1) from a fitting with a Gaussian function. The average size estimated was that expected, in accordance with the synthesis procedure described before [31].

### 3.2. Quantification of Grafted Functional Groups

**Figure 2:**
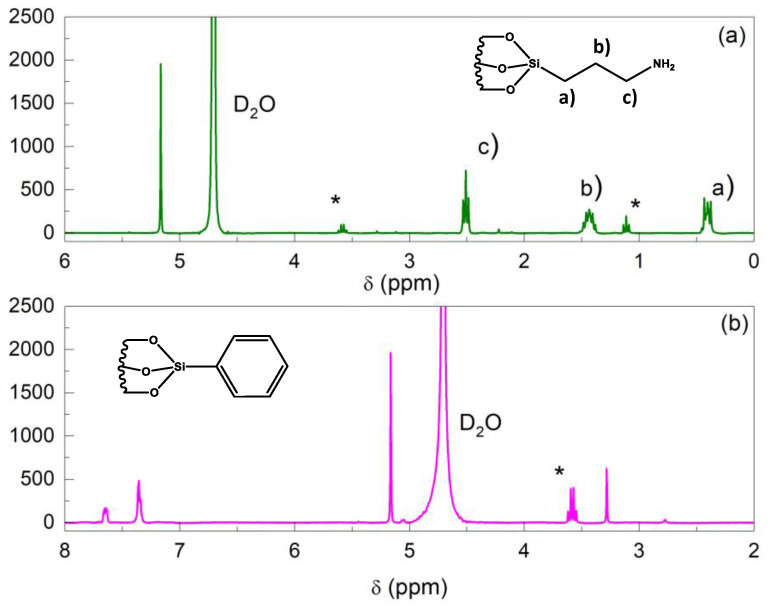
Solution ^1^H-NMR (silica structure destroyed at pH = 13) of functionalized MSN with (**a**) APTES and (**b**) TMPS groups. Peaks are identified with the corresponding functional groups in accordance with chemical structures in the insets. Residual ethanol protons are denoted by (*).

The amount of functional groups in the MSNs was determined using NMR following the procedure previously described [38]. The ^1^H-NMR spectra obtained for both of the modified silicas are represented in Figure 2. All of the peaks have been assigned and associated with specific atom positions in the corresponding structures. At 1.2 ppm and 3.6 ppm, the resonance peaks observed correspond to residual ethanol from washing the MSNs after synthesis.

The integral of the 1,3,5-trioxane (used for calibration) peak at 5.17 ppm was compared to the integral of the protons from the α-carbon to the silicon atom at 0.38 ppm, to estimate the amount of organic APTES molecules grafted onto the surface. Regarding TMPS, the peaks between 8 and 7 ppm were assigned to the aromatic protons (see Figure 2b). The integrated intensity of the peaks from the aromatic ring was normalized to account for its protons.

In both of the modified silica samples, the density of the grafted groups (see Table 2) is lower than the density of silanol in fully hydroxylated silica (~5–7 OH nm^−2^ [39,40,41]). This is the result of the reaction between each APTES or TMPS molecule through one, two or three hydrolyzed ethoxy moieties with the same number of silanol groups of the MSNs’ surface [42]. Nevertheless, the functionalization with TMPS leads to a significantly lower density relative to APTES, possibly due to the bulkiness of the phenyl group turning the adjacent reaction sites inaccessible.

### 3.3. FTIR Analysis of MSNs Loaded with FNB

The modification of the MSNs with APTES or TMPS introduced changes in the infrared spectra of the pristine silica matrix (Figure S1). The band at 1086 cm^−1^ was assigned to the Si–O–C asymmetry stretching vibration and Si–OH stretching vibration [43]. Additionally, a large band was detected between 3600 and 3000 cm^−1^, assigned to hydroxyl groups of adsorbed water (O-H stretching vibrations), and the respective bending modes were detected at 1636 cm^−1^ [44,45].

The modified MSNs_APTES shows additional bands relative to the bare MSNs (see green arrows in Figure S1): (i) a structured band around 2938 cm^−1^ attributed to the C-H stretching vibration [46], and (ii) at 1554 cm^−1^, related to the N-H bending vibrations of the aminopropyl moiety [47]. These bands confirm that the amino-propyl groups of APTES were successfully grafted onto the MSNs.

The presence of the TMPS modifying agent in the silica matrix leads to the appearance of two small bands at 702 and 745 cm^−1^ (see a scale up in the inset of Figure S1), which, in accordance with [48], are assigned to rocking motions of C-H in the phenyl group.

**Figure 3:**
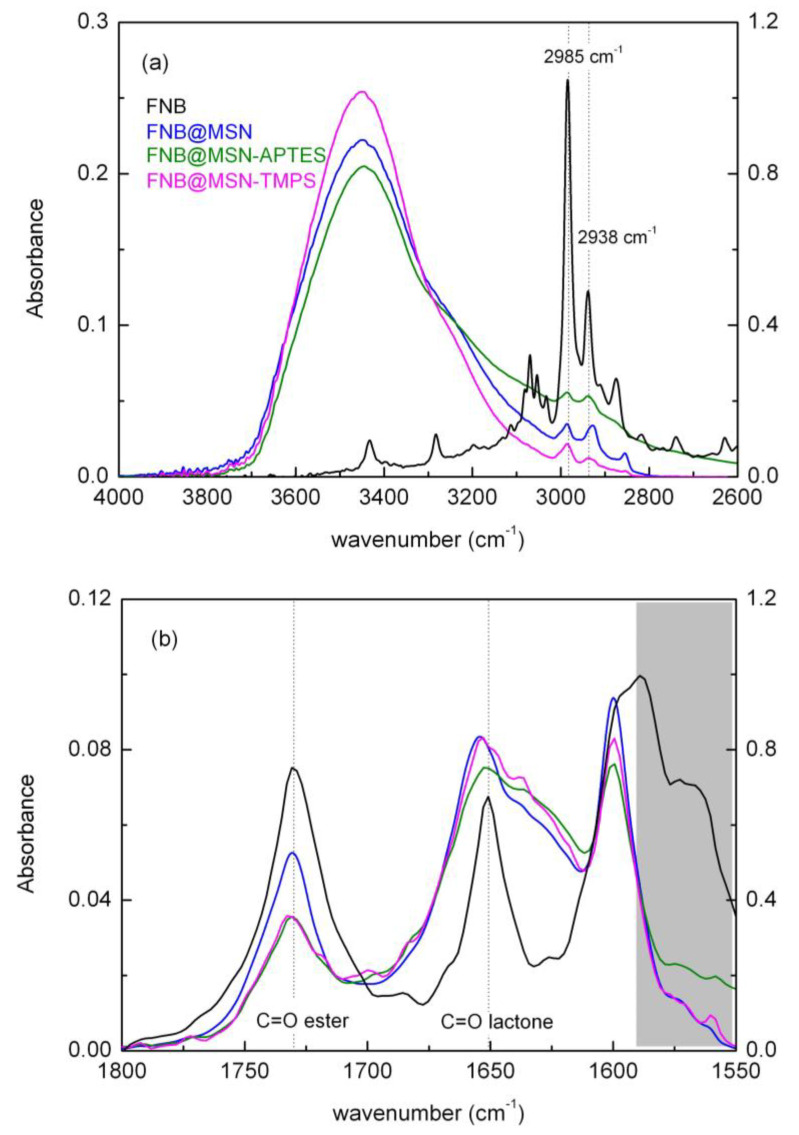
FTIR spectra of FNB@MSNs (blue), FNB@MSNs_APTES (green) and FNB@MSNs_TMPS (pink) in (**a**) 4000–2600 cm^−1^ hydroxyl and aliphatic CH regions and (**b**) 1800–1550 cm^−1^ carboxylic region. See Figure S2 as well, in which the resulting spectra after subtracting the weighted spectrum of the corresponding unloaded MSNs are represented. Spectrum of bulk FNB (black) is displayed on right axis.

To evaluate whether FNB was successfully loaded, the spectrum of the neat crystalline drug was collected and compared with that of FNB loaded into the MSNs (Figure 3). The presence of FNB in the loaded nanoparticles was detected via several small sharp bands, characteristic of the neat drug superimposed to that of the unloaded matrices. The regions due to the C-H and C=O vibrations, located, respectively, between 3800 and 2600 cm^−1^ (Figure 3a) and 1800 and 1550 cm^−1^ (Figure 3b), will now be analyzed in more detail and compared with the spectra of the loaded materials.

Spectral region of 4000–2600 cm^−1^. In the wavenumber region between 3000 and 3100 cm^−1^, crystalline FNB presents multiples weak to moderate bands due to the C-H stretch of the FNB aromatic rings [49,50]. Immediately below this region, three sharp bands are detected, with the ones located at 2986 and 2938 cm^−1^ being assigned to saturated aliphatic C-H stretching vibrations [49,51]. These can be distinguished in the spectra of the FNB-loaded nanoparticles; however, those found for the neat amorphous FNB were slightly broadened and their relative weights had changed [22,52]. Moreover, a close inspection of the 2936 and 2874 cm^−1^ bands of the neat FNB (see Figure S2) reveals a shift to lower wavenumbers upon loading. This could have arisen from amorphization and the building up of interactions with the pore walls’ silanol groups. It has been reported that weak CH···O intermolecular interactions are known to exist in FNB polymorphs linking either aromatic (most stable polymorph I, IIa and IIb [53]) or isopropyl methyl hydrogens (polymorph IIb and III [10]) and ketone oxygen. Upon incorporation into MSNs, this kind of interaction could arise between the CH groups of FNB molecules adsorbed close to the pores’ surface, and -OH from the silica matrices. In the case of the MSN-APTES matrix, this region is also affected by the superposition of the CH from the aminopropyl moiety absorption band (Figure S1).

Around 3500 cm^−1^, a broad band is detected in the loaded matrices’ spectra, also observed in the unloaded matrices, which are absent in bulk FNB. In both the unloaded and loaded matrices, the spectral response is perfectly simulated by two Gaussian functions (see Figure S3) centered at 3455 ± 4 and 3235 ± 4 cm^−1^, which are in good accordance with the wavenumber found by D. Ngo et al. [54] and assigned, respectively, to hydrogen bonding between water molecules and Si-OH hydrogen bonded to water. The absence of bands centered at higher wavenumbers means that neither free water (3540 and 3625 cm^−1^ [55]) nor isolated Si-OH (3740 cm^−1^ [54]) exist in the composites. The normalization of the spectral response for the unloaded and loaded matrices (see Figure S4) shows that the high wavenumber flank remains unaffected upon drug incorporation. Nevertheless, the low wavenumber side is sensitive to the type of matrix, being slightly depleted in FNB@MSN_TMPS, while it broadens in FNB@MSN and even more so in FNB@MSN_APTES, relative to the respective empty matrices’ spectra; this contribution was considered in the overall simulation by a band included at ~3100 cm^−1^ (see blue band in Figure S3). The widening to low wavenumbers could reflect the existence of strong HBs involving FNB, water molecules and silanol/amino groups of the host.

Spectral region of 1800–1550 cm^−1^. The carbonyl stretching region displays two well-defined bands in crystalline FNB, as is shown in Figure 3b, centered at 1728 cm^−1^ and 1650 cm^−1^. These are assigned to the C=O stretching of, respectively, the ester (C=O2-O1) and the ketone groups (C=O4) [52,56]; see Scheme 1 for the identification of these functional groups. A shift to higher wavenumbers, to (1731 ± 1) and (1656 ± 1) cm^−1^, respectively, is observed for the loaded MSNs, together with band broadening relative to the spectrum of crystalline FNB; such modifications are still detected even when the weighted contribution of the unloaded matrices (which exhibit a broad band in this region) is removed from the spectra (see Figure S4). These changes are similar to the ones reported in the literature for amorphous FNB [27,57], acting as first evidence that incorporated FNB exists in the amorphous form. However, in particular for the lactone carbonyl band, although a similar shift is observed for three nanocomposites, it shows a broadening (see Figure 3b and Figure S4) mainly toward the low wavenumber side, being more pronounced in FNB@MSN_TMPS, suggesting the existence of FNB interactions with the holding matrices.

Below the C=O absorption region, crystalline FNB shows a structured band with a maximum at 1599 cm^−1^ associated (Figure S5) with vibrational modes of the aromatic rings (in-plane stretching and deformation of benzene unities) [52]. In the three loaded silicas, a depletion of the low wavenumber shoulder is observed, while the sharp and well-defined 1599 cm^−1^ absorption band prevails, also observed upon neat FNB amorphization [52].

### 3.4. Differential Scanning Calorimetry (DSC)

To evaluate the thermal transformations of the incorporated drug, differential scanning calorimetry (DSC) was used, and the resulting behavior was compared with that of the neat drug and the unloaded matrices. The nanoparticles only show the endotherm signal due to water removal (see Figure S6 and Table S1), whose amount was estimated from the weight loss after calorimetric analysis. Lower water content was found for MSN_APTES, which is not unexpected due to the high density of APTES grafting (see Table 2) that could replace up to three silanol groups, leaving less available sites for water adsorption. However, it should be noted that the terminal amine moieties of APTES must interact more strongly than silanol with water molecules, as inferred by the MSN_APTES calorimetric endotherm that extends to higher temperatures relative to unmodified MSN and also the TMPS-functionalized matrix. In the latter, much less of a density coverage was achieved and, therefore, the water uptake was similar to that of the bare matrix.

Concerning neat FNB, the calorimetric analysis showed that it melts at 81.4 °C, which corresponds to crystalline form I [23,58]. It easily vitrifies by cooling from the melt, even at low cooling rates such as 1 °C min^−1^ [23]; nevertheless, upon heating at 5 °C min^−1^, after the glass to supercooled transition (T_g-on_ = −17.7 °C), it tends to recrystallize with a cold crystallization temperature onset slightly above room temperature ([23], Figure S7).

**Figure 4:**
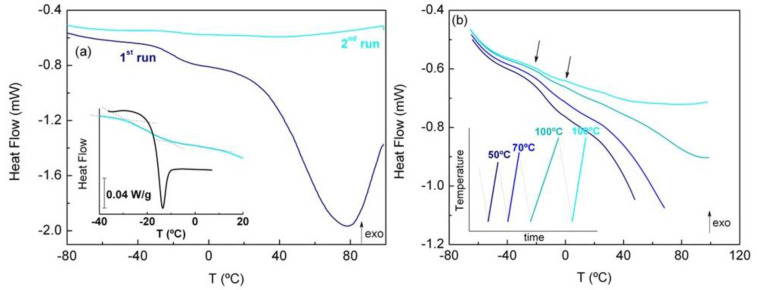
Heating thermograms of FNB@MSNs obtained at 10 °C min^−1^ with the following: (**a**) Two successive cycles with final temperature of 100 °C (water content ~4.9%); inset shows a scale up of the glass transition region for bulk amorphous FNB (black) and hydrated FNB@MSN (light blue). (**b**) Evolution of the glass transition step by varying the final temperature from T_end-1_ = 50 °C to T_end-2_ = 70 °C, T_end-3_ = 100 °C and T_end-4_ = 100 °C; the arrows indicate the onset of each step associated to the bimodal glass transition. The inset shows the schematic temperature vs. time procedure applied.

When FNB is incorporated into unmodified MSN, the thermogram obtained at 10 °C min^−1^ shows the step of the glass transition below −20 °C, and at higher temperatures shows a large exothermal peak related to water evaporation (Figure 4a). This behavior is similar in both FNB@MSN_TMPS and FNB@MSN_APTES; however, in the latter, a small and sharp endothermal peak at 81.2 °C overlaps with the water release signal, likely related to a small FNB crystalline fraction (commented on in the next section). In subsequent cooling/heating scans, no evidence of recrystallization/melting was detected; however, the glass transition step was hardly distinguished.

To understand how water influences the thermal behavior of MSN-incorporated FNB, a gradual drying procedure was applied to a fresh sample of each nanocomposite, consisting of successive cooling/heating cycles to increase the final temperatures (see inset in Figure 4b). Representative thermograms collected upon heating for the FNB@MSNs are shown in Figure 4b. The step associated with the glass transition, clearly observed in the first heating, is progressively depleted upon water removal with a concomitant emergence of a second discontinuity at higher temperatures (the arrows in Figure 4b indicate the respective onsets); this will be commented upon further when analyzing the dielectric results. The onset temperatures of the first step (T_g-on_) are summarized in Table 3.

From Figure 4 and Figure S8, two main characteristics are observed for the three samples: (i) a shift in the glass transition to higher temperatures under drying, and (ii) a significant broadening of the glass transition step relative to the neat drug. Regarding observation (i), this means that the glass transition temperature depends on the hydration level, being lower for higher water contents, caused by water plasticization, as observed for other low-molecular-weight compounds [59,60]. This effect is more striking for the FNB@MSNs, for which a decrease close to 20 degrees was observed, for a sample with a hydration level close to 5% (see the inset of Figure 4a).

Concerning the broadening of the heat flow step (ii), it suggests that, upon loading, the guest drug is able to explore a variety of configurations, ranging from the pore core to the silica surface. Given that all matrices have comparable pore size dimensions, the differences persisting in the glass transition temperature of the nanocomposites in the dried state, i.e., after eliminating the water effect, provide a picture of the drug distribution inside the pores and/or of the drug–silica interactions. This will be discussed in more detail when analyzing the dielectric data.

Additionally, it is clear that the magnitude of the glass transition step decreases when going from the hydrated to the dried state, for each sample. Since the heat capacity is a measure of the configurational degrees of freedom [61], their loss when crossing the glass transition during cooling (as the supercooled liquid becomes frozen) determines the magnitude of the calorimetric step at T_g_. In the supercooled liquid regime, it is expected that the hydrated sample possesses a higher number of degrees of freedom than in the corresponding dried state, as the water molecules decrease guest–guest interactions and prevent, to some extent, the drug adsorption at the pore wall. Indeed, the interaction with the host surface reduces the guest degrees of freedom, and, therefore, the glass transition step during vitrification. Furthermore, the anchoring of a fraction of the drug molecules to the pore walls, leading to the formation of an almost immobile layer, could cause the decrease in the step associated with the glass transition after drying. Ultimately, if the incorporated drug forms a thin monolayer directly interacting with the inner pore wall, it is expected that it will not respond calorimetrically, as found for other drugs loaded on similar silica matrices [62].

For a deeper understanding of the dynamic behavior of FNB loaded in MSNs, around the glass transition, embracing both supercooled and glass states, dielectric relaxation spectroscopy (DRS) was used.

### 3.5. Dielectric Relaxation Spectroscopy (DRS)

Due to the dipolar moment of FNB, DRS can be used for characterizing the molecular motions of FNB in the mesoporous matrices over a large frequency range, from 10^−1^ to 10^6^ Hz. Spectra were collected upon heating from −150 °C up to 150 °C regarding the unloaded nanoparticles, and up to 120 °C for samples with fenofibrate (to avoid the degradation of the guest molecule). The temperature was increased in different steps: from −150 °C to 50 °C in steps of 5 °C and in the remaining temperature range, every 2 °C.

#### 3.5.1. Unloaded Hydrated Matrices

As shown via DSC, water influences the glass transition and hence the dynamical behavior of FNB in the silica matrices. Thus, the water response in unloaded MSNs was prior dielectrically characterized. Each unloaded matrix was submitted to two successive series of isothermal spectra from −100 to 150 °C; the second run was collected after keeping the sample at 150 °C for 20 min to ensure water evaporation. It should be noted that at the end of the chemical modification of the MSNs’ surface, the matrices were submitted to severe dehydration (see Section 2.3), and, thus, the water detected in the first run of the dielectric measurements was water reabsorbed during storage.

**Figure 5:**
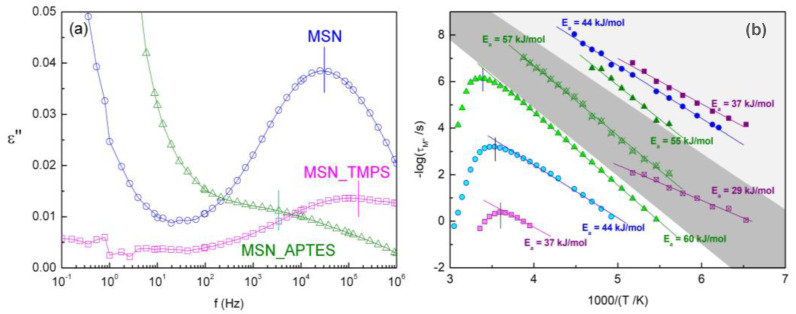
(**a**) Permittivity spectra (ε″) collected at −90 °C for hydrated unloaded MSNs, and (**b**) relaxation map of all of the processes detected in hydrated unloaded matrices (fitting in M″(f)). Straight lines represent the fitting with an Arrhenius function for processes I, I* and II, respectively, in light gray, dark gray and white shadowed regions. An Arrhenius function for process II was only fitted for the low-temperature branch.

Several relaxation processes are identified for each MSN in the hydrated state, located at different temperatures, and they have different relative intensities (see a representative ε″(*f*) spectrum at −90 °C in Figure 5a, Figure S9, in the M″(f) representation). Except for MSN_APTES, the dielectric loss (ε″) becomes negligible in the second-series spectra, reinforcing the assignment of the relaxations detected in the first series to reorientational processes involving water. In the APTES-modified matrix, the persistence of a dielectric response means that there is still some water retained in the silica nanoparticles. The fact that different spectra were obtained for each matrix means, on the one hand, that there was an effective modification of the surface of the nanoparticles, either by TMPS or APTES, and, on the other hand, that such a modification impacted the dynamics of water in the nanoparticles.

To calculate the relaxation times of the water reorientational motions, the imaginary component of the complex electric modulus, M*(*ω*) *= M*′(*ω*) *+ iM*″(*ω*), was analyzed using the corresponding part of a sum of Havriliak–Negami fitting functions (Equation (1)), and a model-independent relaxation time was determined by Equation (2). The obtained relaxation times for all of the detected processes allowed for the construction of the relaxation map, presented in Figure 5b.

The relaxation times (in a logarithmic plot) of the processes in the tested frequency window above −100 °C for the three nanoparticles (hereafter designated as process I) follow a linear temperature dependence described by the Arrhenius Equation (3). Regarding the hydrated unmodified silica, the estimated activation energy is 44 kJ mol^−1^, compatible with the process assigned to reorientations of ice-like water cluster structures found in bulk ice by Johari et al. [63]. Nevertheless, the chemical modification of the MSNs impacts the activation energy, since when silanol groups of the bare matrix are capped by amine moieties (APTES), the activation energy increases, while functionalization with the more hydrophobic (but at a lower coverage) TMPS leads to a decrease in E_a_ (Table 4). This must reflect the extent and surface-directing water hydrogen-bonded (HB) arrangement. For water in porous glass [64], and in non-porous silica nanoparticles [65], a proportionality was found between the activation energy and the number of HBs connecting the clustering water molecules, for an equivalent low-T relaxation process. Concomitantly to the E_a_ increase, the Arrhenian pre-exponential factor, τ_∞_ (see Table 4), decreases, becoming significantly lower than the time corresponding to local non-cooperative relaxation (Debye time, τ ≈ 10^−13^ s) [66]. This indicates that there is some degree of cooperativity between the relaxing dipoles that are involved in process I. The change in both E_a_ and τ_∞_ for the three MSNs agrees with the rationalization proposed in [65] (herein labeled as process 2), and allows for the ordering of the extent of cooperativity of HB water clusters relaxing in the different environments as MSN_APTES > MSN > MSN_TMPS.

As process I shifts to higher frequencies with the temperature increase, a weak relaxation emerges in the low-frequency range of the spectra of functionalized MSNs, hereafter named process I*. The corresponding relaxation times follow an Arrhenius tendency from which the E_a_ and τ_∞_ values were extracted (Equation (3)). The estimated activation parameters are also influenced by the surface functionalization (see Table 4), and, therefore, the underlying relaxation mechanism must involve joined reorientations of the surface chemical units and the interacting water molecules. In the case of hydrophobic MSN_TMPS, water molecules interact preferentially with bare silanol groups, whereas in the more hydrophilic MSN_APTES, the water molecules also interact with the grafted groups. In unmodified MSN, the process is not detected due to a conjunction of different factors: the high intensity of process I, the high dispersion of water molecules in the homogeneous inner pore wall (only silanol groups) and the closeness of the incoming low-frequency side process, hereafter designated as process II; see Figure S10, whereby the individual HN fitting functions for spectra at −90 °C are included, allowing one to visualize the relative positions of process I and I*. Process I* in MSN_APTES is the one still detected after heating up to 120 °C, which is a clear indication that the thermal treatment was insufficient for achieving full dehydration, as previously observed via calorimetric analysis, due to strong APTES–water interactions.

Upon the temperature increasing, process II emerges with a high intensity presenting a singular behavior with temperature in the activation map, whereby the profile is referred to in the literature as being saddle-like [64,67,68,69]. This particular contour results, on the one hand, from the increase in the mobility of loosely bound water with increasing temperature, evolving as a simply thermally activated process (low-T branch). On the other hand (high-T branch), it is due to the contribution of two mechanisms: (i) the removal of nearly free water by a joint effect of temperature increase and the nitrogen flow circulating through the sample cell that drags water molecules out during each isothermal spectral acquisition, and (ii) the slowing down of the reorientational motions of the remaining water molecules, which are more strongly bound to the pore wall.

The 3D representation of M″(f,T) mirrors the concurrence of all of these effects (Figure S11). The temperature dependence of the relaxation times of the low-T branch of process II obeys an Arrhenius law, allowing one to estimate both the pre-exponential factor (τ_∞_) and activation energy, which follow the same trend as in I and I* (respective parameters included in Table 4). The kink point of the saddle-like profile is located at higher temperatures for APTES (T_max_ in Table 4), in agreement with its higher ability to retain water, as mentioned above.

The results reported in this section show how the hydration level and surface modification influence water mobility, even at a low functionalization density, such as the one achieved via TMPS grafting.

#### 3.5.2. Bulk FNB

The molecular mobility of bulk amorphous FNB was probed via dielectric spectroscopy to recognize its signature in the loaded matrices. The drug amorphization was achieved by cooling the corresponding crystalline form from the melt at c.a. 8 °C min^−1^. The dielectric spectra in both of the ε″ and M″ representations were fitted via the superposition of HN functions.

Two main relaxation processes were detected, a dominating process associated with the dynamic glass transition, a designated α-process (visible in the frequency window above ~ −30 °C) and a secondary γ relaxation (emerging at temperatures as low as −110 °C), attributed to the rotation of the C_6_H_4_O_3_(CH_3_)C=O2-O1(CH_3_)_2_ group (Scheme 1), relative to the dihedral angle between two aromatic groups; [22,70] the intramolecular nature of this process was demonstrated according to its low activation energy (close to 30 kJ mol^−1^) and pressure insensitivity by Szklarz et al. [71]. The here-obtained parameters (Table S2) for both α relaxation and the γ-process are similar to the ones reported in the literature [22,71]. A third process is required in the fitting analysis, partially overlapping with the α peak, whose features allow for the identification of it as a Johari–Goldstein (JG) process (β_JG_), as previously suggested in the literature, [70] being pressure-sensitive as a characteristic of a process with an intermolecular nature [71]. The detailed analysis for the bulk FNB is shown in the Supplementary Materials section Dielectric analysis of amorphous bulk FNB.

#### 3.5.3. FNB-Loaded Matrices

**Figure 6:**
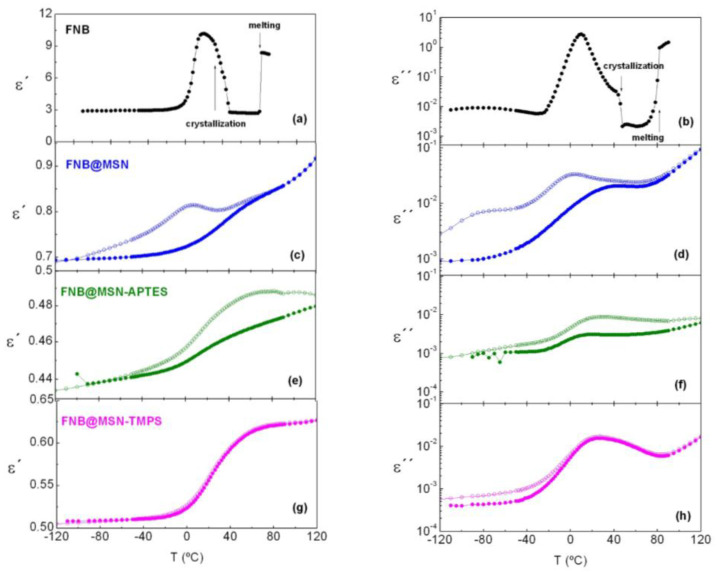
Isochronal representation of ε′ (left panels) and ε″ (right panels) vs. T at *f* = 10^4^ Hz for (**a**,**b**) native FNB, illustrating the effect of cold crystallization and melting, (**c**,**d**) FNB@MSNs, (**e**,**f**) FNB@MSNs_APTES and (**g**,**h**) FNB@MSNs_TMPS; open and filled symbols for hydrated and dried data, respectively.

After FNB incorporation, the dielectric response of the loaded silica MSNs was modified, relative to the unloaded matrices, as exemplified in the isochronal representation displayed at 10^4^ Hz in Figure 6 of the real, ε′ (left side), and imaginary, ε″(right side), parts of the complex permittivity for both the first (hydrated; open symbols) and second (dehydrated; filled symbols) series of spectral collection; data for the neat FNB are included at the top of Figure 6 (black symbols). The comparison of the ε′(T) and ε″(T) traces of the three composites with the ones corresponding to the neat drug, allows for the assigning of the dielectric response observed between 0 and 40 °C to FNB. In the hydrated loaded matrices, the already-characterized water processes are also visible mainly in the low-T region (open symbols in Figure 6); after dehydration, these processes vanish (FNB@MSN and FNB@MSN_TPMS) or become highly depleted (FNB@MSN_APTES), allowing one to better disclose the FNB contribution.

The previous calorimetric analysis showed that amorphous FNB undergoes cold crystallization above 40 °C, and melting close to 80 °C (Figure S14). These phase transitions are also observed in DRS via the sharp decrease (crystallization) and subsequent increase (melting) in both ε′(T) and ε″(T) traces (indicated by vertical arrows in Figure 6a,b). Upon FNB incorporation, no clear signal of melting was found for the studied materials; however, for FNB@MSN_APTES, minor discontinuity was visible in ε′(T) and imperceptible in ε″(T), around 80 °C. This could have originated from the melting of a residual recrystallization of FNB, coherent with DSC analysis (see also Figure S14). It should be noted that FNB recrystallization is observed when it is loaded in silica matrices with larger pore sizes (≥18 nm), with a lower melting temperature for smaller pore sizes [19]. The absence of melting in FNB@MSN and FNB@MSN_TMPS means that FNB has been incorporated within the pores and, furthermore, the pore size (~3 nm) lies below the critical diameter for FNB crystal growth. In the case of FNB@MSN_APTES, the minute endotherm signal at the same neat FNB melting temperature indicated that a rather small fraction was deposited outside of the pores during preparation. After heating up to 120 °C, liquid FNB should enter inside the pores, as no evidence of either recrystallization/melting was detected.

In the following sections, the dielectric response of FNB incorporated into each MSN sample will be analyzed. Despite a severe drying procedure prior to FNB loading, the loaded matrices easily reabsorbed water (explaining why two series of spectra were collected). In the first series (hydrated), the fitting analysis was only carried out for spectra acquired between −110 °C and ~30 °C (ending T, depending on the material), as above this temperature, the sample dielectric response continuously changes due to progressive water release (the dielectric cell is not isolated, and it is uninterruptedly submitted to nitrogen flux).

**Figure 7:**
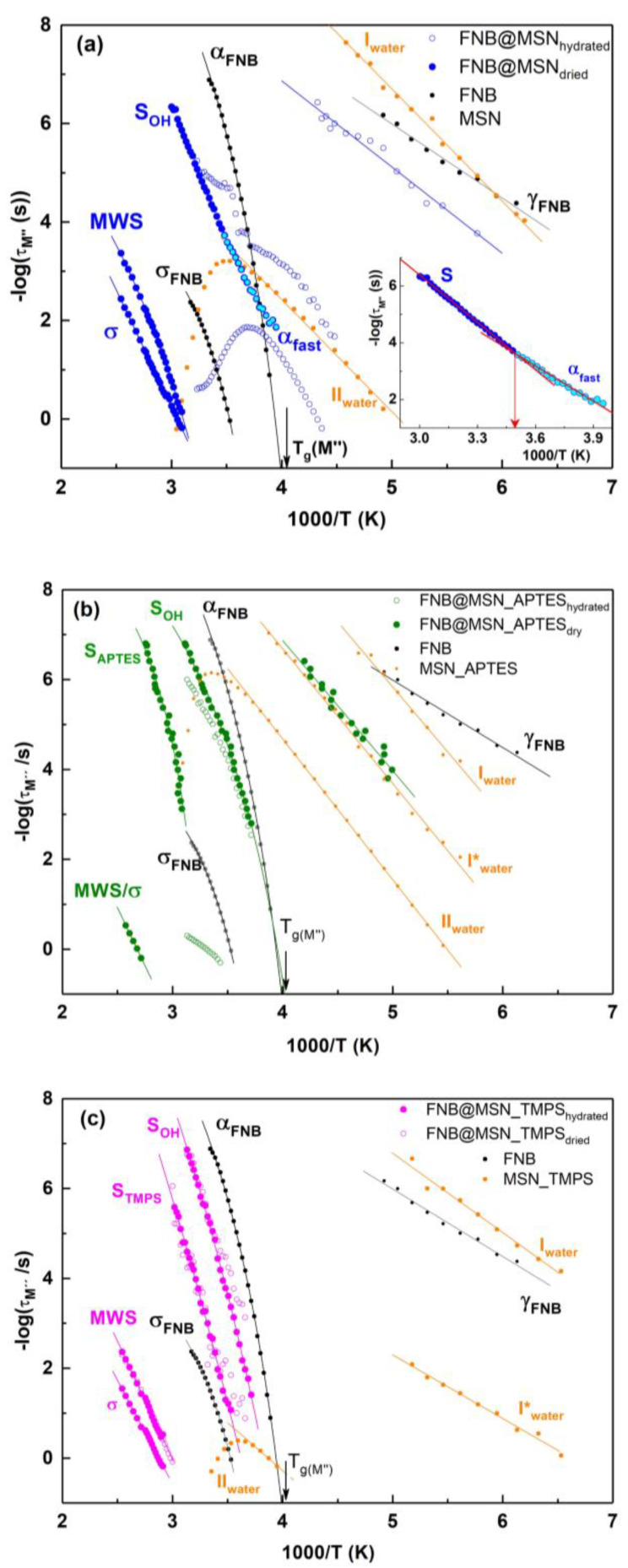
Relaxation maps of (**a**) FNB@MSN, (**b**) FNB@MSN_APTES and (**c**) FNB@MSN_TMPS. Open symbols correspond to hydrated series data and filled ones correspond to dried series. In each figure, the relaxation times of different processes detected in the corresponding unloaded matrix (orange symbols) and those present in bulk FNB (black symbols) are included. Solid lines are the fitting functions with Arrhenius or VFTH estimated for each process. The inset in (**a**) is a scale up of the second run (dried) of the bending at ~12.7 °C (1000/T = 3.5 K^−1^) from α-fast to the S-process compared to α_FNB_-trace (see text).

Non-functionalized MSN loaded with FNB. The isothermal spectra collected for FNB@MSN, both hydrated (first run) and dehydrated (second run), were analyzed using a sum of HN functions (Equation (1)). Representative fitting curves and a summary of obtained parameters are included in ESI (Figure S15 and Table S3). The temperature dependence of the obtained relaxation times for all of the detected processes are represented in Figure 7a (hydrated—open symbols; dehydrated—filled symbols), with neat FNB and unloaded MSN data included as black and orange symbols, respectively. For the process detected at the lowest temperatures, logτ(T) evolves linearly, and so, it can be fitted by the Arrhenius equation. An activation energy of 33 kJ mol^−1^ was obtained with a pre-exponential factor of 1.4 × 10^−14^ s. This relaxation process could be attributed to either a modified γ-process or to process I observed for hydrated unloaded MSN (orange circles in Figure 7a). Since after dehydration this process is absent, it is most probably assigned to the water relaxation. Nevertheless, relaxation involving reorientational motions of the (C=O2-O1) ester group according to the attribution of the secondary γ in neat FNB should not be discarded. Given that the drug is a proton acceptor species [57,72] and the matrix has both a proton donor and acceptor silanols [73], it is reasonable to assume that part of the FNB C=O groups interact via hydrogen bonding with the silanol groups of the MSNs’ surface. In this sense, it is important to note that the FTIR analysis provided evidence of strong interactions involving the Si-OH groups (Figure S3a). After dehydration, with the water release, more adsorption sites become free to interact with FNB molecules, thus strengthening the drug–host interaction, and, therefore, strongly depleting the amplitude of the unbonded FNB γ dipolar motion, impairing its dielectric detection in the second run.

At higher temperatures, an intense and broad process emerges on the low-frequency side, which is adequately simulated by a sum of two relaxations (see illustration of the individual fitting functions in Figure S15a,b). The assignment of these processes becomes clear via the analysis of their temperature dependencies in the relaxation map (Figure 7a). The more intense process follows saddle-like temperature dependence, vanishing after dehydration. It is assigned to process II, previously observed in the hydrated unloaded matrix (included in Figure 7a as orange symbols). The T-dependence of the high-frequency process is curved, bending off at ~10 °C, a temperature at which water starts to be removed from the material (confirmed by the coincidence with the temperature corresponding to the τ maximum of the FNB@MSN process II). This behavior can be rationalized as in the lowest T-range: water is relaxing with FNB, enhancing the drug mobility relative to the neat drug (black symbols), resulting in a plasticizing effect. From here, τ(1/T) follows the temperature dependence of the bulk FNB α-relaxation (black symbols), and, since water is removed as the temperature increases, a shift in the relaxation times toward slower values occurs. For the highest temperatures, the τ(1/T) dependence becomes linear. This last temperature dependence is similar for that of the dehydrated FNB@MSN (filled blue symbols in Figure 7a), with both traces superimposing in the high T-range. The latter is unequivocally resolved over a large T-range, due to the absence of water process II in the second run (see isotherm at 0 °C in Figure S15c).

The continuous deviation in the relaxation time mirrors the decreasing FNB mobility, concomitant with water release. This may have originated from a progressive adsorption of FNB to the silica pore walls at sites previously occupied by water molecules, resulting in a dynamical hindrance relative to the neat drug, as the relaxation times of this process lie on the low-frequency/high-temperature side of the bulk α-process. Moreover, the temperature dependence after water removal is Arrhenius-like (activation parameters in Table 5), in line with the features exhibited by a surface (S) process found in low-molecular-weight glass formers adsorbed at the silica inner pore walls [74,75,76]. A close inspection of the T-dependence of the relaxation times reveals a bending at 12.7 °C (see inset in Figure 7a) near/above the region where the α_FNB_-trace is crossed. Additionally, the temperature dependence of the magnitude of the electrical modulus (ΔM) reveals a crossover occurring at a similar temperature (Figure S16), reinforcing the alteration in the overall dynamics, probably caused by the freezing of the surface population. The latter could have originated from the second high-T step detected in the thermogram (Figure 4b).

Below this crossover region, the relaxation times become lower than those of the bulk α_FNB_-process. This acceleration of the relaxation rate may be interpreted as a manifestation of finite size effects of a distinct population being localized in the pore core. Indeed, when a material is confined to dimensions of the order of the cooperative rearranging regions (in the framework of the Adam–Gibbs model) [77], their relaxation times can undergo an acceleration effect with a decrease in the glass transition temperature (chapter 6 in [27,76]), an effect compatible with the reduction in T_g-on_ observed via DSC (Table 3). Consequently, this low-T relaxation can be designated as α_fast_. Ultimately, if the pore diameter is lower than the correlation length associated with the size of the cooperatively rearranging regions, the T-dependence converts from non-Arrhenius (continuous growth of cooperativity) to Arrhenius (constant or absent cooperativity) [78]. Apparently, the T-dependence of the detected α_fast_ process is linear, although to confirm the Arrhenian behavior, the scanned range should be extended to even lower frequencies.

The enhancement in the relaxation rate at low temperatures and the lowering of the onset of T_g_ (onset of the first step in Figure 4b) found for FNB incorporated into MSN were also reported for the same drug loaded in nanoporous AAO membranes with higher pore diameters [19]. While crystallization was observed in the AAO membranes, it was avoided in the tested MSN matrices with pore sizes ~3 nm. Above 60 °C, an additional process emerges, which will be further discussed when analyzing the behavior of the three systems.

Functionalized MSNs loaded with FNB. It has already been shown that surface functionalization affects the dynamics of water processes and, therefore, it is expected to also influence the molecular mobility of the guest drug. This will be evaluated in the present section by comparing the dielectric response of matrix-functionalized composites with that of an unmodified matrix. At first glance, the main differences are as follows: (i) the detection of a single process in the low-T region (whose ill definition only makes possible the fitting in the second series of FNB@MSN_APTES); (ii) the lack of the water saddle-like process at intermediate temperatures for both matrices (in spite of comparable water contents), meaning that loosely bound water is absent in these samples and (iii) the detection of a relaxation process clearly comparable to the FNB α_bulk_-process.

The assignment of the different processes detected in functionalized MSNs will be made according to the temperature dependence of their relaxation times included in the activation maps in Figure 7b,c (Figures S17 and S18 illustrate the fitting procedure of isothermal spectra for a set of representative temperatures). With regard to the low-T relaxation in FNB@MSN_APTES (Figure 7b) observed in the second run, the overlapping of the respective log(τ(T)) trace with that observed for the MSN_APTES unloaded matrix allows one to undoubtedly assign it to process I* of water. This confirms the strong tendency of the APTES-modified MSNs to retain water, differently from the other composites, in which water was merely reabsorbed after FNB loading. In the first run, FNB@MSN_APTES shows a clear process above the glass transition of bulk FNB (the corresponding relaxation times are included in Figure 7b as open circles), although affected by the progressive water release during acquisition data upon heating. In the second series of measurements, an additional relaxation is visible at higher temperatures. For FNB@MSN_TMPS (Figure 7c), two processes are also needed to deconvolute the M″ spectra in both hydrated and dry states. Their T-dependence of relaxation times is very similar for both hydrated and dry series of measurements coherent with a low water uptake.

The relaxation times of the two processes, observed in the second run of both modified MSNs, exhibit a non-Arrhenius temperature dependence that can be described by the VFTH fitting function (fitting parameters are summarized in Table 5). The emergence of the processes at temperatures higher than the T-range at which α bulk FNB is observed reflects a hindrance of the underlying molecular motions, and so they can be identified as surface processes. To explain their origin, it should be considered that two types of guest–host interactions can arise in the modified MSNs: (i) between silanol and FNB, and (ii) between grafted APTES or TMPS groups with FNB. The former is expected to exist in modified and unmodified composites, giving origin to a surface process common to three systems (hereafter designated as S_OH_). The position of the relaxation times of this S_OH_-process in the modified MSNs relative to the one observed in FNB@MSN reflects a weakening of the silanol–FNB interactions due to the presence of APTES or TMPS. The extrapolation of the VFTH fitting functions to τ = 100 s [79] provides an estimate of the glass transition temperature, −28 °C and −23 °C, respectively, for APTES and TMPS; considering the corresponding dielectric glass transition temperature for the bulk FNB (−25.5 °C; see Table S2), the T_g-dielec_ was thus estimated, keeping the same relative order found for the calorimetric T_g-onset_. For the case of FNB incorporated into an APTES-functionalized matrix, both DSC and DRS concur disclosing a dynamically accelerated population relative to the neat drug at low temperatures (decreased T_g_), however to a lower extent, relative to what was found for FNB@MSN. The fact that the shape parameters that simulate the FNB@MSN_APTES dielectric spectra remain almost constant for all of the temperature ranges suggests that the same kind of cooperative rearrangement is being probed, being less (low T) or more (high T) influenced by the surface.

On the other hand, the second process (located at slower relaxation rates relative to the S_OH_-process) may have originated from interactions involving not only the silanol groups but also the grafted groups. Besides the Si-OH···O=C interactions, N-H···O=C interactions in the APTES matrix or π-π stacking between the TMPS matrix and FNB aromatic units could also arise. This anchoring of the same FNB molecule at two sites of the pore wall will further constrain the guest dynamics. Two reasons can sustain this hypothesis: (i) the lengthening of APTES and bulkiness of TMPS moieties relative to Si-OH and (ii) the high flexibility of the FNB aliphatic chains allowing the guest molecule to adopt different conformations, as already reported for this drug [52]. Both (i) and (ii) favor the binding via a non-covalent interaction at multiple sites, which is already known to occur in other guest–host systems [80]. The slower relaxation rate of the MSN_APTES surface process and the near absence of a calorimetric response (blue DSC curve in Figure 4a) indicate FNB-APTES as being the strongest guest–host interaction.

The present investigation clearly demonstrates that DRS analysis allows disclosing several relaxation processes which are in the origin of the widening and multimodal characteristics of the glass transition region observed via DSC (remember the DSC curves in Figure 4b and Figure S8).

#### 3.5.4. Conductivity of the Loaded Samples

At temperatures above reorientational dipole motions, DRS mainly probes the migration of charge carriers. The conductivity behavior gives an insight on how the modifications in the confining media reflect the long-range charge mobility.

**Figure 8:**
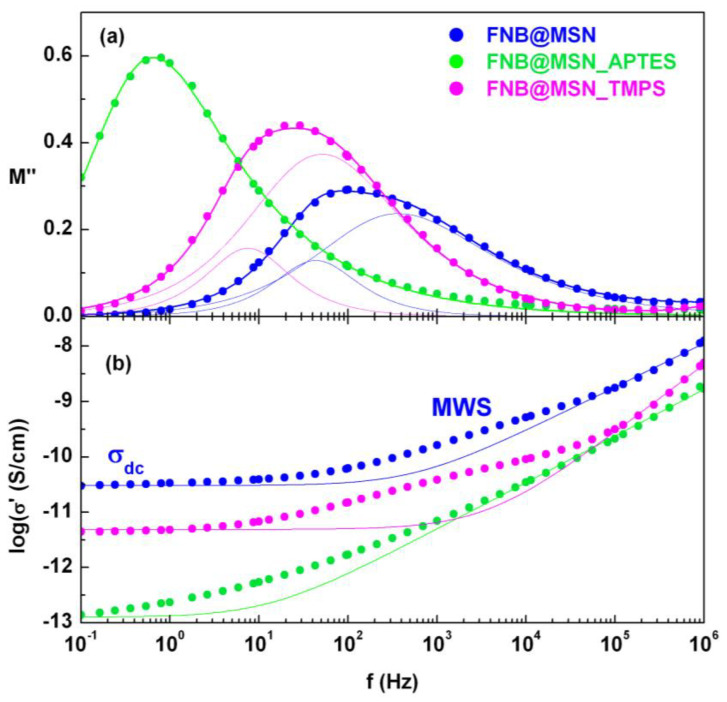
Isothermal spectra at 120 °C of the FNB-loaded nanoparticles (**a**) in the M″(*f*) representation, with the spectra deconvoluted in two HN functions (MWS and σ) for FNB@MSN and FNB@MSN_TMPS (thin lines), and only one HN function for FNB@MSN_APTES (overall fit shown as thick lines); (**b**) σ′(*f*) spectra for the three composites, with the isotherm σ′(f) predicted by the Jonscher law for FNB@MSN (solid lines).

The second run of the isothermal measurements of FNB@MSN revealed a highly asymmetric peak in the modulus representation (Figure 8a) and multiple regimes in the conductivity spectra, σ′(*f*) (Figure 8b). The latter shows three different regions: a dc plateau at low frequencies (1), which bends off to a frequency dependent regime (2), distinct from the ac high-frequency behavior (3). The dielectric response at intermediate frequencies must have originated from a Maxwell–Wagner–Sillars (MWS) effect [81], as expected in heterogenous systems in which charges accumulate at interfaces (with discontinuities in the dielectric properties) [82]. The absence of this effect in bulk FNB (Figure S19a) supports the attribution to MWS relaxation in the composite. The effect is clearly seen in Figure 8b, with a strength that can be quantified by the difference between the experimentally measured conductivity (symbols) and the isotherm predicted by the Jonscher law [83]. The latter just takes into account diffusive (at low frequencies, at which the plateau emerges) and semi-diffusive (ac frequency response at high frequencies) charge transport mechanisms, according to ($\sigma^{\prime }\left(\omega \right)=\sigma_{0}\left[1+{\left(\omega /\omega_{cross}\right)}^{s}\right]$), in which σ_0_ is the direct current conductivity (estimated in the frequency-independent regime), *s* is a material and temperature-dependent parameter (0.5 ≤ *s* ≤ 1) and ω_cross_ is the angular frequency at which the *dc* plateau changes to an *ac* regime. In the FNB@MSN M″(*f*) spectra, both *dc* conductivity and the MWS process emerge as the corresponding peaks overlap in this temperature range, causing the abovementioned asymmetric M″(*f*) peak. This peak can be deconvoluted in two HN functions: one at the lowest frequencies (fitted with shape parameters α_HN_ = β_HN_ = 1 as expected for a pure *dc* conductivity process (chapter 3 in [27])), and another function which takes into account the interfacial MWS contribution (see the thin solid lines in Figure 8a and Figure S19b as examples). The same effect is observed in the TMPS functionalized silica (Figure 8a).

For MSN_APTES, the MWS contribution is mainly visible, as confirmed by the shape parameters (α_HN_ = 0.85 and β_HN_ = 0.5), with the conductivity contribution lying in the low flank of the frequency window. The absence of a well-defined *dc* plateau reflects the hindrance to long-range charge migration imposed by the presence of the APTES moiety. The observed behavior for the functionalized matrices loaded with FNB results from the balance between the degree of grafting—higher for APTES, increasing MWS contribution—and the amount of incorporated drug—higher for TMPS, increasing the conductivity contribution.

The temperature dependence of the MWS and σ_dc_-processes is well described by either an Arrhenius or a VFTH plot, depending on the sample. The corresponding fitting parameters are presented in Table 5.

Differently to what happens in bulk FNB, for which the Debye–Stokes–Einstein correlation is obeyed (inset of Figure S12), pointing to a coupling between translational (σ_dc_) and reorientational dipolar motions (τ_α_), for FNB loaded in the nanoparticles, it is not possible to test the proposed relationship, due to the large temperature gap between the dipolar processes and the emergence of *dc* conductivity.

### 3.6. Release Kinetics

**Figure 9:**
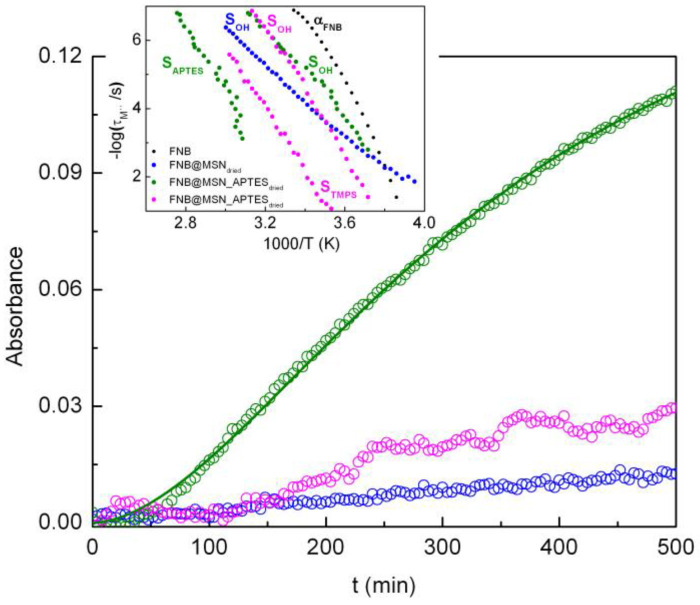
Release profile measured from absorbance at λ = 289 nm of FNB from MSN (blue), MSN_APTES (green) and MSN_TMPS (pink). The data corresponding to the FNB@MSN_APTES sample were modeled using the Hill equation (Equation (6)) with *A*_max_ = 0.17, *γ* = 1.77 and *A*_50%_ = 32,751 min^1.77^ as the fitting parameters. Inset: relaxation map including only surface processes (colored symbols) and α of bulk amorphous FNB (black markers).

Samples previously investigated via DRS were used in the FNB release tests. To analyze the release profile, absorbance at λ = 289 nm, in which the FNB spectrum shows a maximum, was represented as a function of time (Figure 9). The masses used in these tests were very similar (0.00113 ± 0.00003 g), facilitating the direct comparison of the results (see the Experimental section for the specific details of the drug delivery assays).

At the initial stages (up to ~50 min), almost no FNB was released in any of the samples. Considering that most of the water was removed during the dielectric measurements, this time lag is likely related to the slow diffusion of FNB from within the pores of the matrix, driven by the intrusion of the releasing medium into the nanoparticles, explaining the absence of a burst release. This supports the previous indication that FNB molecules are mostly located near the pore walls, rather than being in the core or outside of the pores.

After this initial step, the increase in the absorbance was very pronounced in the MSN_APTES sample, while for MSN and MSN_TMPS the release rate of the loaded FNB was significantly slower. The FNB release profile from MSN_APTES can be described using the empirical Hill model (see ref. [84] and references therein), which adapted to absolute values of absorbance reads:

(6) $$A(t)=\frac{A_{max}t^{\gamma }}{A_{50\%}+t^{\gamma }}$$

where *A*_max_ is the maximum absorbance attained (corresponding to a certain value of dissolved drug), *A*_50%_ is the time required to reach 50% of *A*_max_ and γ is a sigma factor. The Hill fitting function well describes the very slow FNB release profile from MSN_APTES (solid line in Figure 9—fitting parameters presented in the caption).

Although only a small fraction of FNB was released at the end of the 8 h for all of the composites (Table 6), the way in which the drug is released depends on the matrix, with the larger difference observed for MSN_APTES. The amount of FNB released from MSN_APTES at the end of the 8 h is about ten times larger than that from the unmodified MSNs. This behavior can be rationalized considering the relaxation times estimated from the dielectric analysis of the dried nanocomposites (i.e., series 2). The FNB molecules involved in the S_OH_-process are expected to be released first (see a close-up of the activation map of the surface processes depicted in the inset of Figure 9). In relation to this process, the FNB relaxes more slowly in the unmodified matrix, causing the lower FNB release rate. On the other hand, the differences observed in the drug release profiles of the two functionalized silica matrices indicate that, in addition to guest mobility (where the relaxation times are comparable), another parameter must be involved. This is probably associated with topological differences on the inner surface of the pore. The type and density of the grafted groups affect the architecture of the pore wall, altering the homogeneity of the adsorbed FNB layer. Indeed, as previously mentioned (Table 2), the density of the grafted groups is not enough to uniformly cover the pore wall surface. These inhomogeneities can weaken the adsorption of FNB to the silanol groups relative to the adsorption occurring in high-density and homogeneously silanol-covered unmodified MSNs, ultimately determining the release rate of the FNB population that leaves the pores first.

## 4. Conclusions

Fenofibrate (FNB) was incorporated into spherical mesoporous silica nanoparticles (MSNs) with external diameters of ~50 nm and pore diameters of ~3 nm. The tested MSNs were either unmodified or modified via the grafting of APTES or TMPS groups (with 1.93 and 0.60 molecules nm^−2^, respectively).

The presence of grafted groups in the MSNs has a noticeable effect on the dynamics of adsorbed water, as observed via dielectric analysis. This allowed for the distinction of three relaxation processes associated with the following: (i) reorientational motions in ice-like clusters (process I), (ii) joined reorientations of the surface chemical units and the interacting water molecules (process I*) and (iii) the relaxation of loosely bound water (process II).

After FNB loading, calorimetric, infrared spectroscopy and dielectric analysis showed that the drug was stabilized in the amorphous state, proving that the pore size was below the critical nucleus diameter for FNB crystallization.

Broad glass transition was observed via DSC (for the three composites), assigned to the loaded FNB drug. Moreover, the glass transition onset (T_g,ons_) slightly shifted to lower temperatures for FNB@MSN and FNB@MSN_APTES, while it increased for FNB@MSN_TMPS. Calorimetric glass transition was sensitive to readsorbed water, mainly for FNB@MSN. DRS confirmed this sensitivity to water content, as the FNB relaxation rate slowed down upon water removal. After dehydration, the mobility at temperatures around the calorimetric glass transition still revealed an accelerated dynamic. The evolution of the respective relaxation times with temperature showed a kink to a more constrained process, assigned to surface silanol groups interacting with FNB molecules (S_OH_-process). In the functionalized matrices, a correspondent S_OH_-process was detected, although it exhibited higher mobility due to a weakening of the silanol–FNB interaction caused by the presence of the grafted groups. Additionally, a high-temperature surface process was detected, associated with FNB interacting mainly with the APTES and TMPS capping groups. Furthermore, the existence of guest–host interfaces was found in the beginning of the MWS process, detected in all composites, affecting the conductivity response.

The drug delivery profiles showed no initial burst release. Instead, drug release was only detected after a significant time interval (~50 min). FNB is released faster from the APTES-functionalized matrix which is interpreted in terms of the higher molecular mobility of the FNB population associated with the S_OH_-process, and heterogeneities in the surface coverage imprinted by the grafted groups.

The work presented here allowed a number of key parameters to be correlated with FNB release behavior, enabling a more rational design of mesoporous silica nanoparticles for drug amorphization and delivery.

## Figures and Tables

**Table 1:** Drug loading conditions.

Sample	FNB Loaded (% *w*/*w*) *	Available *V_p_* (cm^3^)	V_FNB_ (cm^3^) **	Pore Volume Occupied by FNB (% *v*/*v*)
MSN	22.8	0.095	0.035	37
MSN_APTES	22.8	0.096	0.036	37
MSN_TMPS	33.3	0.081	0.050	62

* %FNB mass fraction in the sample; ** volume estimated for crystalline form I of FNB.

**Table 2:** APTES and TMPS concentration on the MSNs calculated from ^1^H NMR spectra.

Sample	mmol g^−1^ MSNs	Modifying Molecules/nm^−2^
MSN_APTES	3.14	1.93
MSN_TMPS	0.98	0.60

**Table 3:** Onset of the glass transition temperature (T_g-on_) values of FNB in MSN, MSN_APTES and MSN_TMPS under successive cooling/heating cycles with different final temperatures; water content was estimated from the weight loss after DSC runs.

Sample	T_g-on_ (°C) Heating 1 T_end-1_ = 50 °C	T_g-on_ (°C) Heating 2 T_end-2_ = 70 °C	T_g-on_ (°C) Heating 3 T_end-3_ = 100 °C	Water Content (%)
FNB@MSNs	−23.5	−21.5	−21.5	4.1
FNB@MSNs_APTES	−20.9	−18.6	−19.8	5.7
FNB@MSNs_TMPS	−14.7	−12.5	−9.4	2.0
FNB	T_g-on_ (°C) = −17.7			

**Table 4:** Activation parameters of all processes found in unloaded silicas. The relaxation times of process I and I* were fitted with Equation (3).

	Process I		Process I*		Process II		
	E_a_ (kJ mol^−1^)	τ_∞_ (s)	E_a_ (kJ mol^−1^)	τ_∞_ (s)	E_a_ (kJ mol^−1^)	τ_∞_ (s)	T_max_ * (°C)
MSN	44	8.0 × 10^−19^	--	--	44	2.9 × 10^−12^	9.0
MSN_APTES	55	4.7 × 10^−21^	57	2.1 × 10^−19^	60	6.3 × 10^−18^	21.8
MSN_TMPS	37	1.8 × 10^−17^	29	1.1 × 10^−10^	n.a.	n.a.	4.2

* Temperature of the log(τ) maximum of the saddle-like profile taken directly from the data.

**Table 5:** Estimated parameters of the Arrhenius fit (Equation (3)) and the VFTH fit (Equation (4)) to the relaxation times for all of the detected processes in the dehydrated samples. Data corresponding to bulk FNB have been included for comparison. All relaxation times were extracted from the fitting of the M″ spectra.

Sample	Process	Function	τ_0_ (s)	E_a_ (kJ mol^−1^)	B (K)	T_0_ (K)
FNB	γ	Arrhenius	3.2 × 10^−14^	28.7		
	α	VFTH	4.0 × 10^−15^		1598	205
	σ	VFTH	1.3 × 10^−6^		635	273
FNB@MSN	α_fast_	Arrhenius	7.7 × 10^−19^	79.4		
	S_OH_	Arrhenius	8.2 × 10^−24^	106.5		
	MWS	VFTH	1.4 × 10^−9^		2395	204
	σ	Arrhenius	3.6 × 10^−15^	90.5		
FNB@MSN_APTES	S_OH/_α	VFTH	8.7 × 10^−13^		1508.8	198.6
	S_APTES_	VFTH	2.1 × 10^−11^		801.8	276.4
	MWS/σ	Arrhenius	1.5 × 10^−14^	98.8		
FNB@MSN_TMPS	S_OH_/α	VFTH	1.5 × 10^−20^		4978.2	151.9
	S_TMPS_	VFTH	1.7 × 10^−23^		8846.7	107.2
	MWS	Arrhenius	1.7 × 10^−16^	100.7		
	σ	Arrhenius	5.1 × 10^−14^	88.5		

**Table 6:** Release data obtained via UV-Vis: mass of sample (*m*_sample_); weight fraction of FNB loaded in MSNs (*FNB*_loaded_); mass of FNB in MSNs (*m*_FNB_); moles of FNB in MSNs (*n*_FNB_); mass of MSNs (*m*_MSN_); moles of FNB in the MSNs per gram of nanoparticles (*n*_MSN_); moles of FNB released after 8 h per gram of MSNs (*n*_rel_); mole fraction released after 8 h relative to loaded FNB (*n*_released_).

Sample	*m_sample_* (g)	*FNB_loaded_* (wt%)	*m_FNB_* (g)	*n_FNB_* (mol)	*m_MSN_* (g)	*n_MSN_* (mol g^−1^)	*n_rel_* (mmol g^−1^)	*n_rel_* (mol)	*n_released_* (%)
FNB@MSN	1.11 × 10^−3^	22.8	2.53 × 10^−4^	7.01 × 10^−7^	8.57 × 10^−4^	8.18 × 10^−4^	2.36 × 10^−3^	2.02 × 10^−9^	0.29
FNB@MSN_APTES	1.13 × 10^−3^	22.8	2.58 × 10^−4^	7.14 × 10^−7^	8.72 × 10^−4^	8.18 × 10^−4^	2.17 × 10^−2^	1.89 × 10^−8^	2.65
FNB@MSN_TMPS	1.16 × 10^−3^	33.3	3.86 × 10^−4^	1.07 × 10^−6^	7.74 × 10^−4^	1.38 × 10^−3^	1.60 × 10^−2^	1.24 × 10^−8^	1.15

## Data Availability

Not applicable.

## Supplementary Materials

The following supporting information can be downloaded at https://www.mdpi.com/article/10.3390/pharmaceutics15061624/s1. References [22,23,70,71,79,85,86,87,88,89] are cited in the Supplementary Materials.
