# Supplementary material for: Understanding Fenofibrate Release from Bare and Modified Mesoporous Silica Nanoparticles
Source: Pharmaceutics. 2023 May 30;15(6):1624. doi: 10.3390/pharmaceutics15061624 (PMC10301214; doi:10.3390/pharmaceutics15061624)
Supplement: Supplementary file 1 [file pharmaceutics-15-01624-s001.zip › pharmaceutics-2384167-supplementary.pdf]

Understanding fenofibrate release from bare and modified mesoporous silica nanoparticles

Giorgia Figari[1], José L. M. Gonçalves[1], Hermínio P. Diogo[1], Madalena Dionísio[2], José Paulo Farinha[1,*] and María Teresa Viciosa[1,*]

[1] Centro de Química Estrutural, Complexo I, Instituto Superior Técnico, University of Lisbon, Avenida Rovisco Pais, 1049-001 Lisbon, Portugal

[2] LAQV-REQUIMTE, Department of Chemistry, NOVA School of Science and Technology, Universidade Nova de Lisboa, 2829-516 Caparica, Portugal

[*] Correspondence: teresaviciosa@tecnico.ulisboa.pt (J.P.F.); farinha@tecnico.ulisboa.pt (M.T.V.)

Table of Contents

S1. FTIR ………………………………………………………………………………………….. 2

S2. DSC ………………………………………………………………………………………….. 6

S3. Unloaded MSNs: confined water ……………………………………………………….. 9

S4. Dielectric analysis of amorphous bulk FNB ………………………………………….. 12

S5. FNB incorporated in non-functionalized MSNs ……………………………………….. 17

S6. FNB incorporated in functionalized MSNs ……………………………………………. 20

S7. Transport properties …………………………………………………………………….. 22

S8. References ………………………………………………………………………………….. 23

S1. FTIR

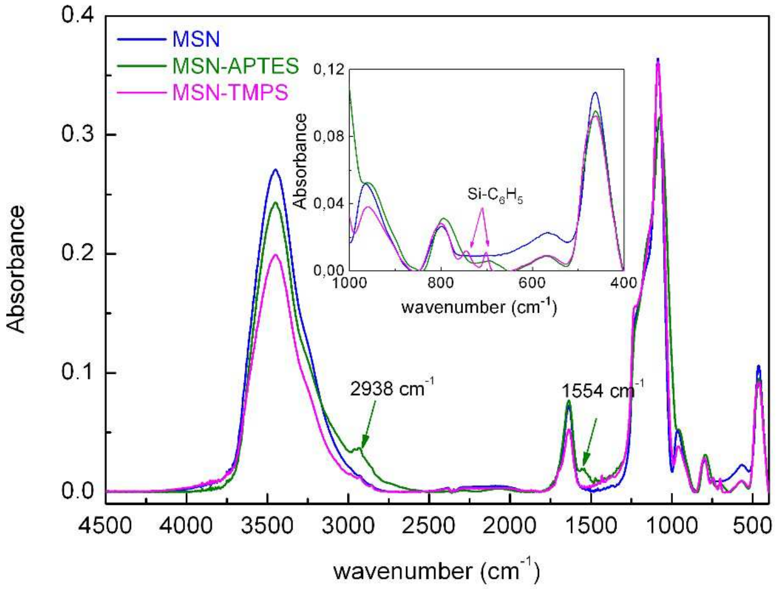

Figure S1. FTIR spectra obtained for three MSN, unmodified (blue), functionalized with APTES (green) and functionalized with TMPS (pink). Green arrows indicate the principal bands where modifications introduced by APTES are detected (2938 cm^{-1} and 1554 cm^{-1} associated to CN and NH respectively). Inset: enlarge region showing the bands assigned to TMPS functional.

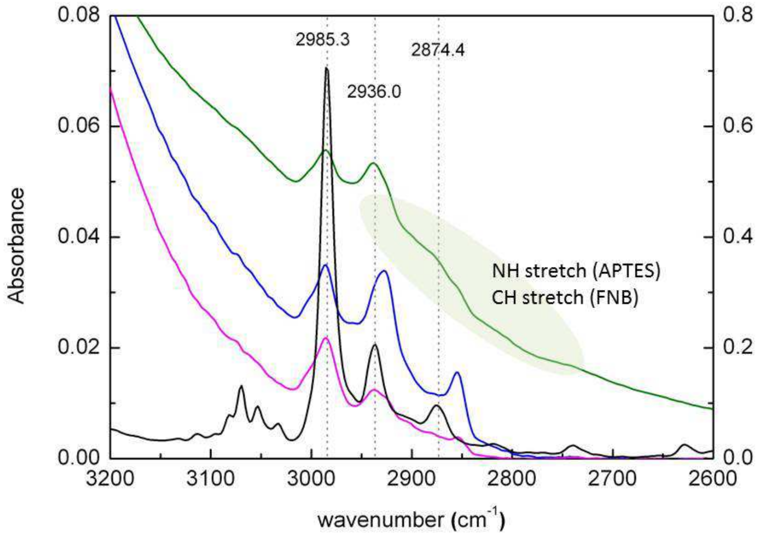

Figure S2. FTIR spectra of FNB (black line, right axis) and loaded MSNs (colored lines, left axis) in the high wavenumbers range.

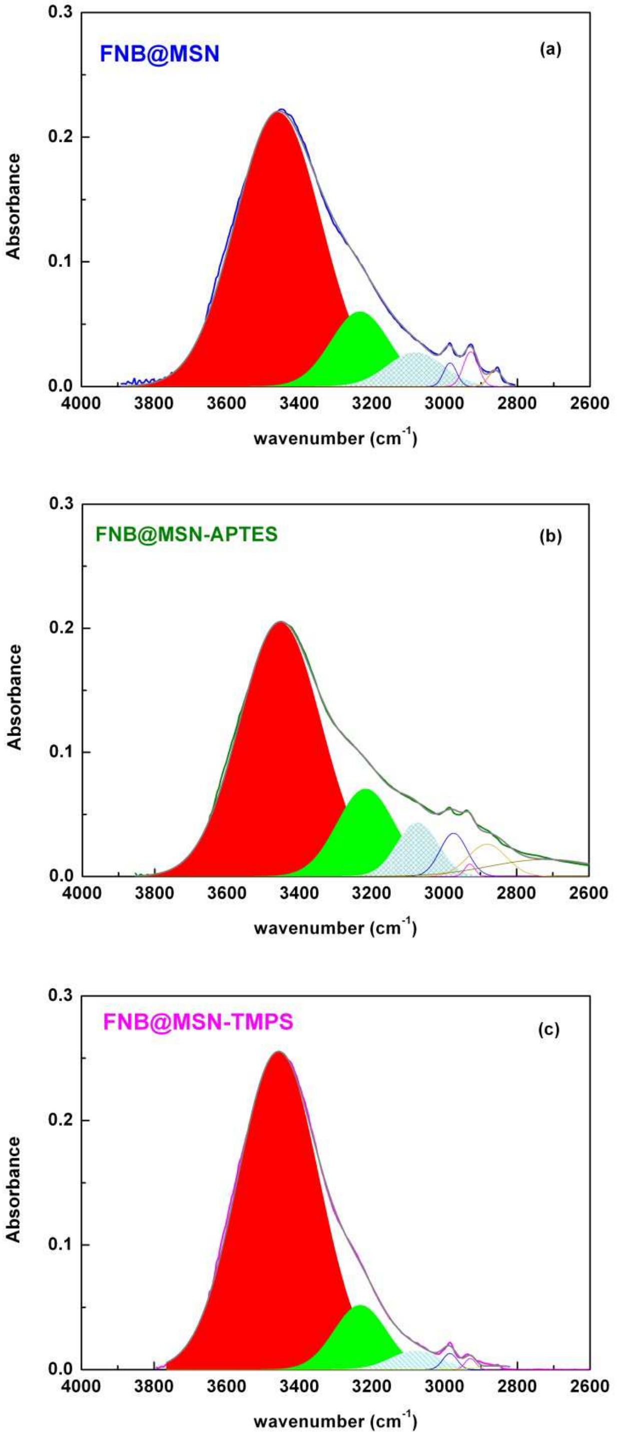

Figure S3. FTIR spectra of FNB loaded MSNs in the high wavenumbers range. Individual gaussians functions used to deconvolute the spectra are included; red band at 3450 ±4 cm^{-1} and green band at 3235 ±4 cm^{-1} correspond to medium-strong HB and strong HB respectively. Band centered around 3100 cm^{-1} shows intensity highly dependent of the matrix.

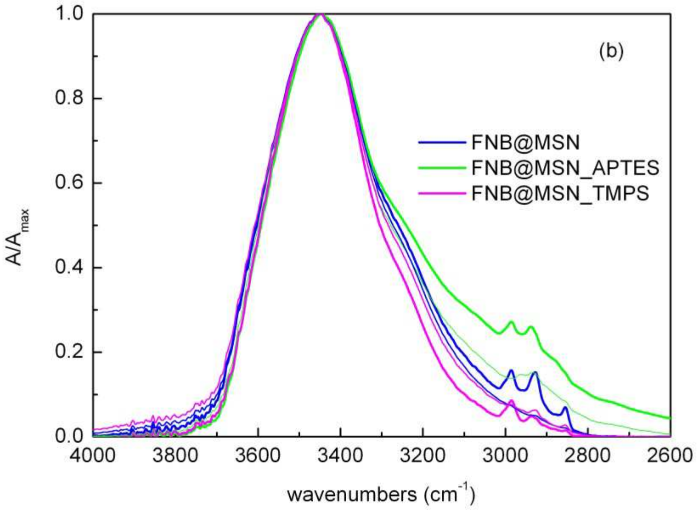

Figure S4. Absorbance spectra normalized to the maximum at 3450.6 cm^{-1} for unloaded and loaded matrixes (thin and thick lines respectively).

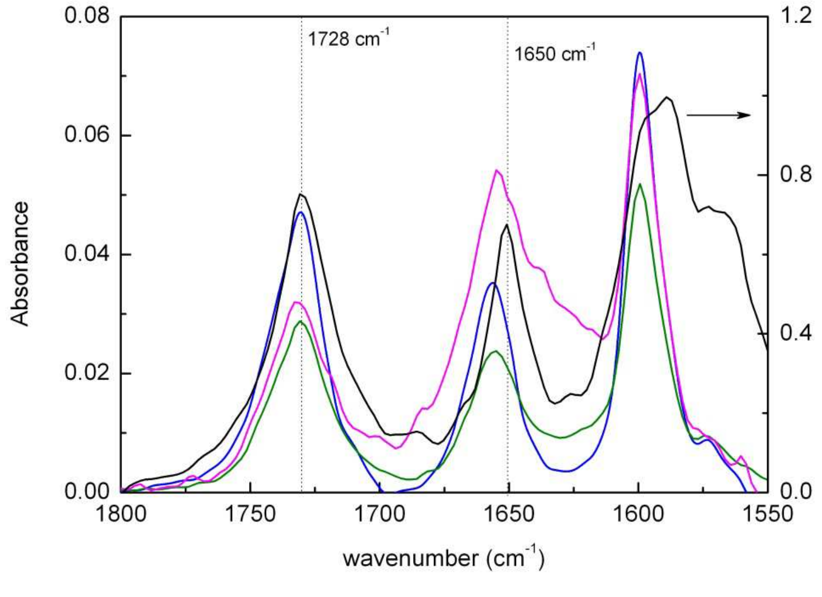

Figure S5. FTIR spectra in two different regions of: FNB@MSNs (blue), FNB@MSNs_APTES (green) and FNB@MSNs_TMPS (pink); the contribution of the corresponding silica fraction has been subtracted from the measured spectra. Spectrum of bulk FNB (black) is displayed in right axis for comparison.

S2. DSC

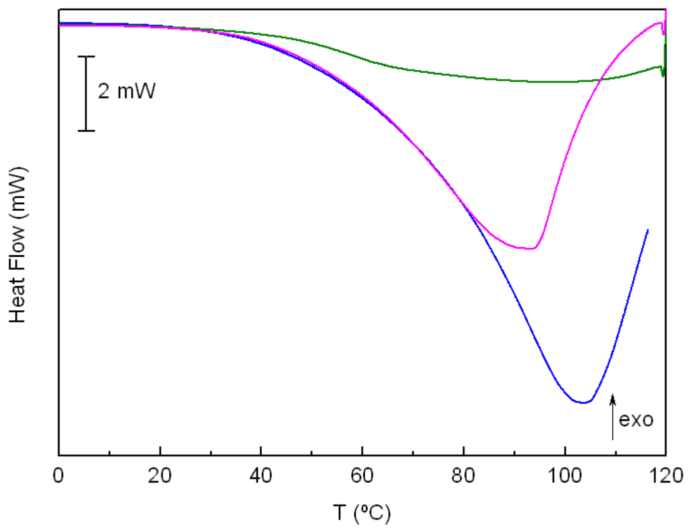

Figure S6. Thermograms of unloaded nanoparticles obtained on heating at 10 °C min^{-1}.

Water content of unloaded nanoparticles was estimated from the loss weight between samples equilibrated at room temperature and: (i) after drying in the oven at 140 °C during 2 hours and (ii) by heating up to 120 °C at 10 °C min^{-1} in the calorimeter. From next table, it can be seen that both methods provide similar water loss for each nanoparticles.

Table S1. Percentage of water loss estimated by drying in the oven or by drying in the calorimeter.

	**ΔM (%) oven**	**ΔM (%) DSC**
**MSN**	26	23
**MSN_APTES**	10	7
**MSN_TMPS**	24	22

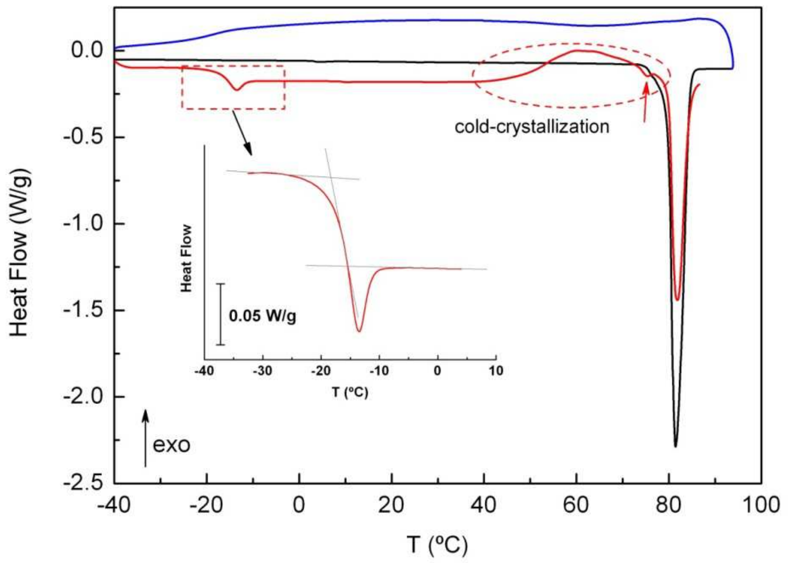

Figure S7. Thermograms of bulk FNB obtained at 5 °C min^{-1}: black, heating of the sample as received; blue, cooling after melting, and red, heating from the glassy state. Inset: enlarge the temperature region of the glass transition on heating thermogram. Data reported in reference 1.

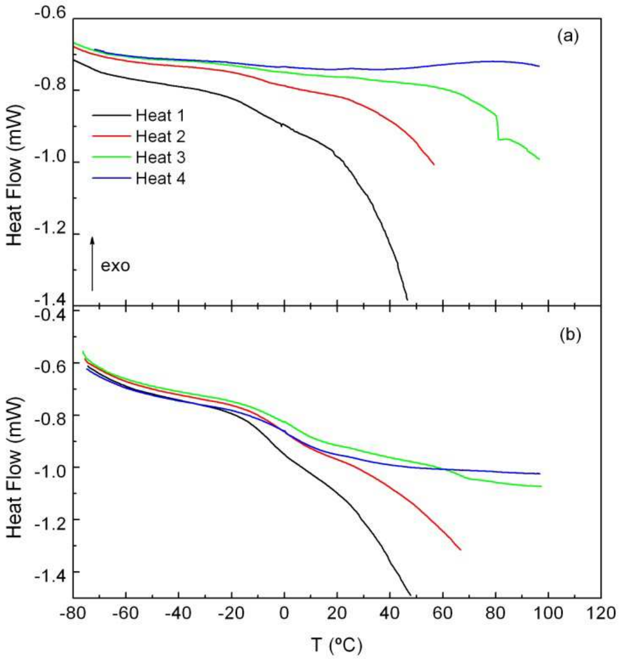

Figure S8. Thermograms of a) FNB@MSN_APTES and b) FNB@MSN_TMPS samples obtained on successive heating scans at 10 °C min^{-1} with different final temperatures: black for T_{end} = 50 °C, red for T_{end} = 70 °C, green for T_{end} = 100 °C and blue for T_{end} = 100 °C.

S3 . Unloaded MSNs: confined water

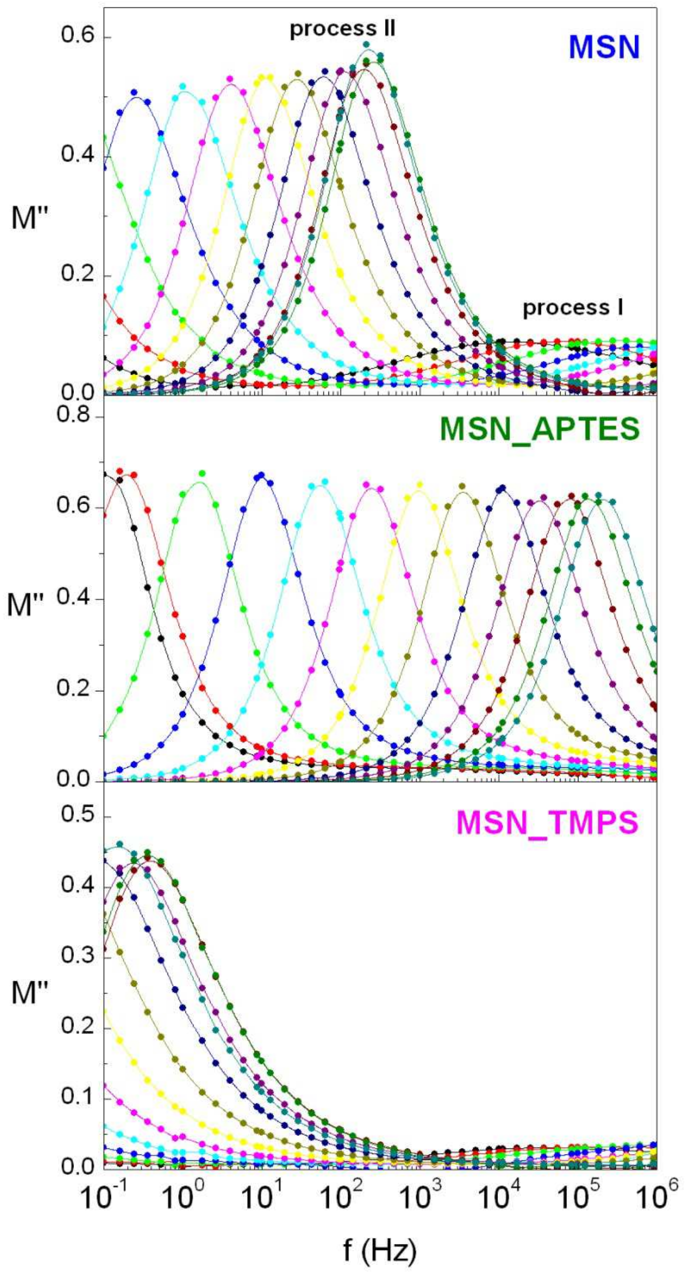

Figure S9. Isothermal M´´ spectra of unloaded matrixes for temperatures from -100 to 0 °C every ten degrees.

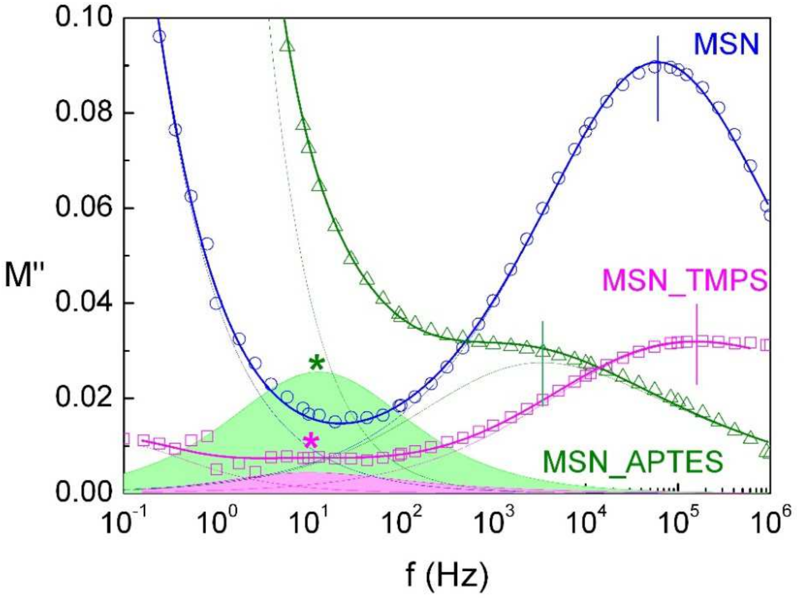

Figure S10. M’’ spectra collected at -90 °C for hydrated unloaded MSNs. Thin lines represent the individual HN fitting functions and the thick lines the overall fit of dielectric data. Straight vertical segments and asterisks indicate the frequency position of process I and process I* respectively.

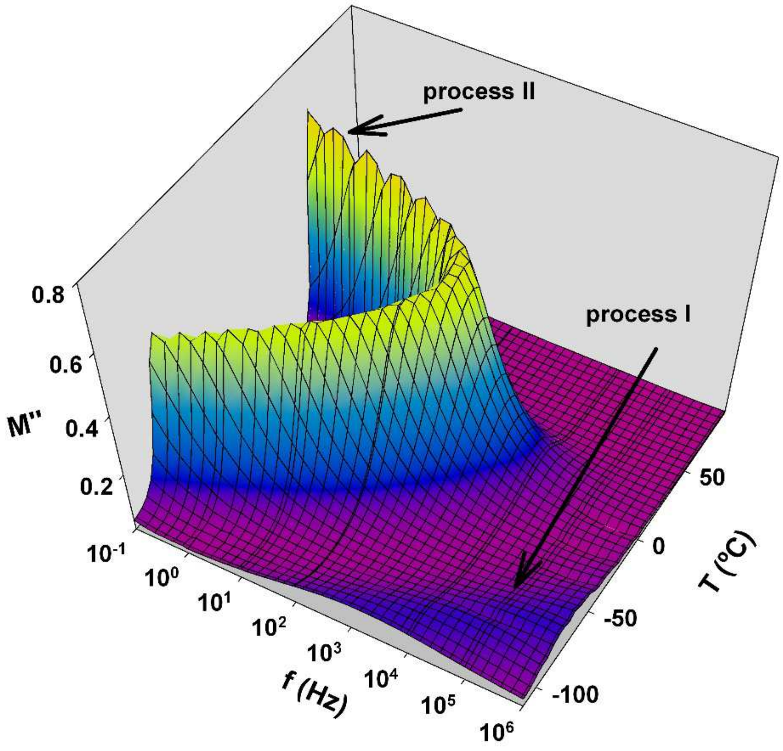

Figure S11. 3D plot of imaginary part of complex permittivity as a function of frequency and temperature for hydrated unloaded MSNs.

S4. Dielectric analysis of amorphous bulk FNB

Few mg of the as received crystalline FNB was sandwiched between two gold-plated electrodes. Two silica spacers of 50 μm was introduced to guarantee the sample's thickness after melting. The crystalline powder was then heated at 10 °C min^{-1} up to 100 °C monitoring the dielectric response at six selected frequencies (10^1, 10^2, 10^3, 10^4, 10^5 and 10^6 Hz). The ε′(T) trace shows a suddenly increases at ~76 °C independent of the frequency, indicating the FNB melting. After readjusting the sample′s thickness at room temperature, it was re-heating again to 100 °C. Then, it was cooled down to -110 °C at *c.a.* 8 °C min^{-1}. Both ε′(T) and ε′′(T) traces doesn't exhibit any signal of recrystallization, otherwise, put in evidence the supercooled and posterior glassy entrance with further cooling. Isothermal spectra were collected from -110 to -50 °C, every 5 degrees, and from -48 to 90 °C, every 2 degrees. This procedure results in an equivalent heating rate of 0.74 and 0.4 °C min^{-1} respectively.

The analysis of the ε′′(f) isothermal spectra was done by the sum of HN fitting functions. Besides the clearly visible γ and α relaxations, during the analysis was necessary to introduce a third function in between. Small differences in the values of HN parameters of α and γ comparing with reported data by Sailaja *et al.* [2] must be assigned to the introduction of the third process in the fitting procedure (see Table ESI 2).

The characteristic relaxation times obtained for each process are represented in Figure ESI 12. As expected, an arrhenius temperature dependence and a VFTH behavior are found for γ and α relaxations (see Table ESI 2 for respective fitting function parameters). The glass transition temperature estimated from DRS data results from the extrapolation of the VFTH fitting function to τ = 100 s [3] leading to the value of -22.6 °C.

Regarding the intermediate process, it was hypothesized that it could be a Johari-Goldstein secondary relaxation, associated to the motion of the molecule as a whole [4,5]. The relaxation times of this JG process has been identified with the relaxation time of the primitive process in the coupling model (CM) [6]. In this framework, the next relationship has been proposed:

$$\tau_{JG}(T) \approx \tau_0(T) = t_c^n[\tau_\alpha(T)]^{1-n} \qquad \text{Equation 1}$$

where τ_0 is the primitive relaxation time of the CM, the coupling parameter $n = 1 - \beta_{KWW}$, τ_α is the relaxation time of the Kohlrausch-Williams-Watts (KWW) stretched exponential function (defined in the time domain) and t_c is a time characterizing the crossover from independent to cooperative fluctuations found to be close to 2×10^{-12} s for molecular glass formers [7]. From the parameters of the HN function of

the α process, and using the transformation proposed by Alegría *et al.* [8] ($\alpha_{HN}\beta_{HN} = \beta_{KWW}^{1.23}$), the β_{KWW} exponent was obtained: β_{KWW} = 0.65. The τ_{JG} obtained by Equation 1 was 1.6x10^{-3} s (at the T_g(DRS) = -22.6 °C), that it was included in the relaxation map as an orange star. The proximity with the relaxation times estimated from the spectra analysis allows identifying this process with a secondary β_{JG} as previously suggested in references [9,10].

Table S2. Shape HN fitting parameters from the fitting of isothermal spectra of ε´´ and M´´, temperature range of all processes analyzed in bulk amorphous FNB. For secondary γ relaxation, the activation energy (E_a) and τ_0 obtained from the Arrhenius fit of the temperature dependence of its relaxation times; for the α process, the VFTH fitting parameters are included. Additionally, the extrapolated T_g (at τ = 100 s), the activation energy at T_g, and the fragility index.

		α_{HN}	β_{HN}	ΔT (°C)	$E_a(T_g)$ (kJ mol^{-1})	-log(τ_0 (s))	B (K)	T_0 (K)	T_g (°C)
ε´´	γ	0.27±0.01	1	[-110,-70]	28.7	3.2x10^{-14}			
	β_{JG}	0.35±0.02	1	[-40,-16]					
	α	0.98	0.60	[-18,34]	458	7.9x10^{-14}	1373	211	-22.6
M´´	γ	0.28±0.02	1	[-110,-70]	28.7	3.2x10^{-14}			
	β_{JG}	0.35±0.02	1	[-40,-18]					
	α	0.87±0.03	0.6→0.42	[-20,26]	458	4.0x10^{-15}	1598	205	-25.2
	σ	1	1	[10,48]		1.3x10^{-6}	635	273	
ε´´	γ				32.67$^{(a)}$/28.2$^{(b)}$	2.5x10$^{-14(a)}$			
	β				77.0$^{(b)}$				
	α$^{(a)}$	0.98	0.57			3.0x10^{-14}	D=7.04±0.2	209±1	-22.6

(a) Data taken from reference 2.

(b) Data taken from reference 10.

The conductivity contribution in the ε´´(f) spectra observed above -8 °C was fitted with the term $-i\sigma/\omega^c\varepsilon_0$ with ε_0 the vacuum permittivity ε_0 and *c* a fitting parameter describing the broadening of the relaxation time distribution for the dc conductivity. The *c* parameter was found to be equal to 1, characteristic of pure dc conductivity. To further comparison with data of loaded nanoparticles, also the spectra in M´´

were analyzed by the same procedure. In this case, to the conductivity contribution observed in ε''(f) corresponds a Debye-peak ($\alpha_{HN} = \beta_{HN} = 1$) whose maximum is clearly defined for temperatures above 10 °C (see for instance Figure ESI 13).

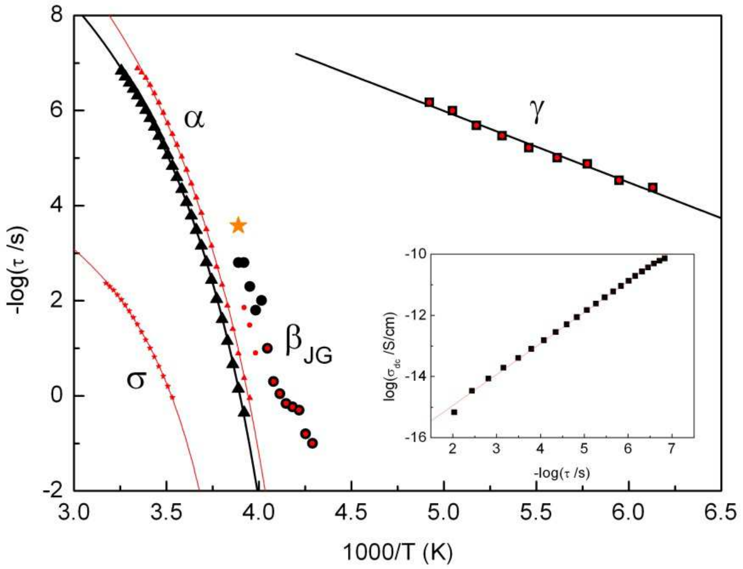

Figure S12. Relaxation map of bulk amorphous FNB: black symbols obtained from the fitting of ε´´(f) and red symbols obtained by the fitting of M´´(f). The yellow star indicates the JG relaxation time estimated from the Coupling Model (Equation 1). Inset: log-log representation of the σ_{dc} and τ_α obtained from the fitting of the ε´´(f) spectra between -8 and 34 °C; linear trend was fitted by log(σ_0) = -16.97± 0.05 and slope = 1.01+ 0.01.

In the temperature range where conductivity and α relaxations are simultaneously detected, it was represented log(σ_{dc}) *vs.* log(τ_α) (see inset of Figure ESI 12). The linear trend is fitted with slope equal to 1.01±0.01 that in accordance with DSE relationship ($\sigma_{dc}(T) \propto 1/\tau_\alpha(T)$) the translational motions associated to conductivity and the orientational molecular motions controlling the structural relaxation are fully coupled; these results agree with the ones provided for bulk FNB at ambient pressure [10].

At 46 °C, the cold-crystallization of the sample is noted in ε´´ by a decrease in the dipolar relaxation strength as well as in the conductivity tail as consequence of the blocking of molecules at the positions of the crystal network. Data of spectra above 46 °C were not considered in this manuscript and will be presented elsewhere.

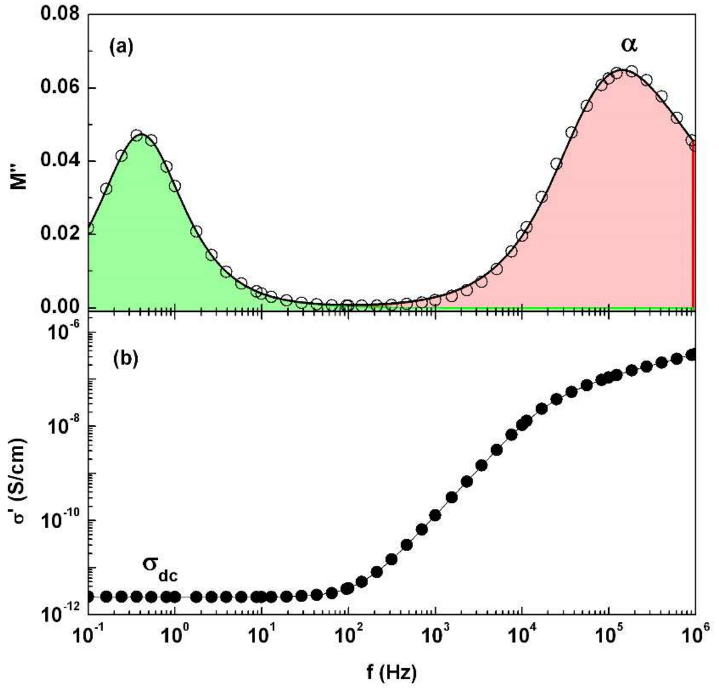

Figure S13. Isothermal spectra of bulk FNB at 14 °C. the low frequency peak in M′′ corresponds to pure σ_{dc}. Solid lines in (a) are the HN fitting functions (imaginary part) used to describe the overall spectrum.

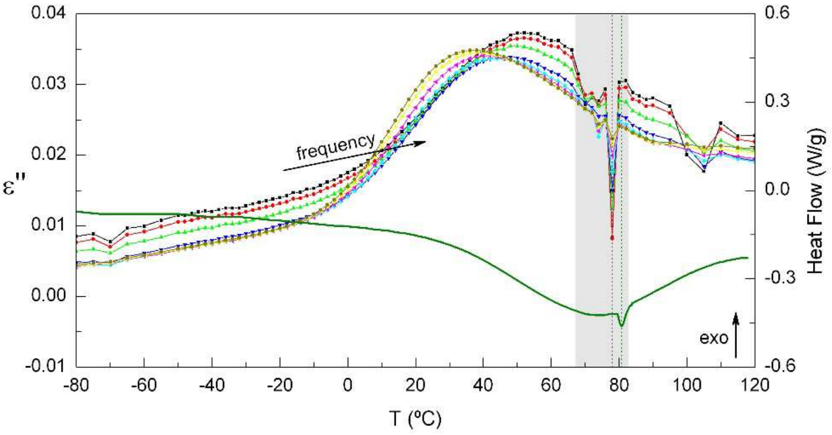

Figure S14. For FNB@MSNs_APTES: isochronal representation of ε´´ *vs.* T at frequencies between 10^6 and 10^5 Hz in left axis (data measured in the isothermal mode); heat flow thermogram obtained on heating at 10 °C min^{-1} of a fresh sample, in right axis. Grey rectangle indicates the region in which a small fraction of FNB melts.

S5. Fenofibrate incorporated in non-functionalized mesoporous silica nanoparticles (FNB@MSN)

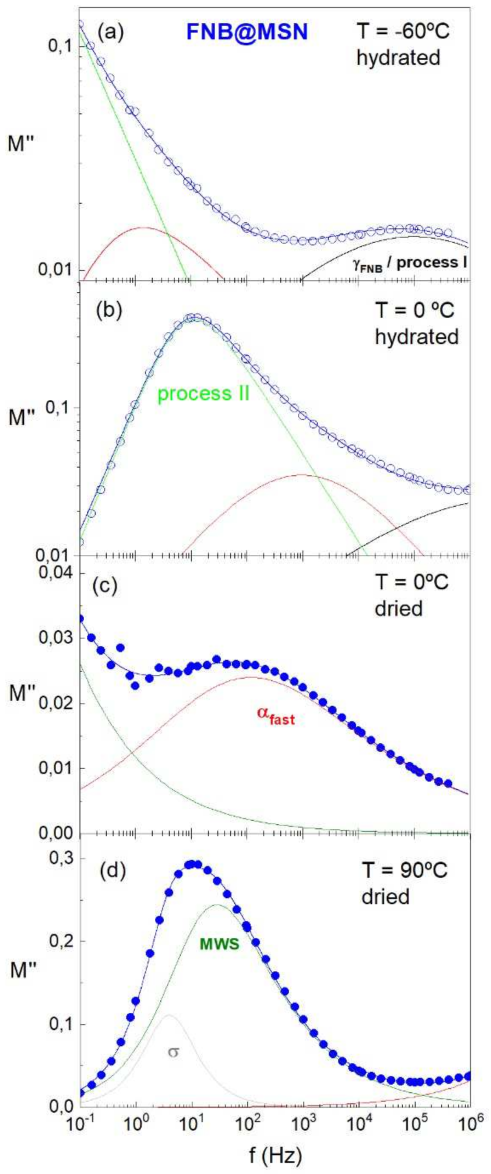

Figure S15. Representative isothermal spectra M´´ *vs.* f of the sample FNB@MSN, blue circles; dotted lines represent the HN individual fitting function (imaginary part), and blue line is the overall fitting curve.

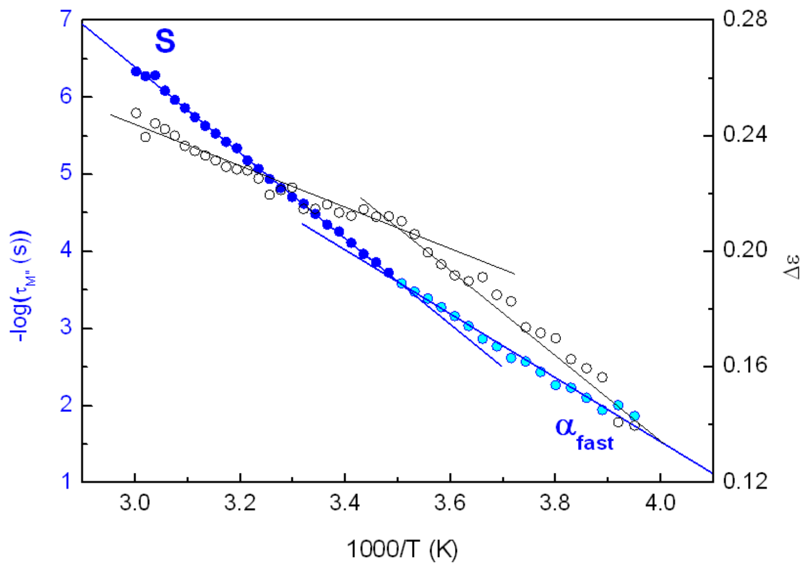

Figure S16. -logτ (left axis) and Δε (right axis) *vs*. 1/T of the S/α_{fast} process detected in dehydrated FNB@MSN. The straight lines put in evidence the existence of a crossover temperature in both parameters.

Table S3. HN shape fitting parameters of the different relaxations observed in loaded nanoparticles. ΔT indicates the temperature range in which each relaxation has been well-defined and considered for posterior analysis of the relaxation times.

	series	process	ΔT (°C)	α_{HN}	β_{HN}
**FNB@MSN**	hydrated	Low T	[-100,-42]	0.32±0.02	1
		$\alpha \rightarrow S_{OH}$	[-50,36]	0.51±0.04	1
		Saddle-like	[-44,36]	0.93±0.02	0.58±0.07
	dried	α_{fast}	[-20,14]	0.36	0.69±0.01
		S_{OH}	[14,60]	0.40±0.02	0.61±0.06
		MWS	[50,120]	0.63±0.06	0.70±0.1
		σ	[50,120]	1	1
**FNB@MSN_ APTES**	hydrated	α / S_{OH}	[-4,46]	0.39±0.02	0.55±0.06
		MWS / σ	[20,48]	1	0.41
	dried*	--	--	--	--
**FNB@MSN_TMPS**	hydrated	S_{OH}	[0,46]	0.38±0.03	1
		S_{TMPS}	[0,60]	0.5±0.11	1
		MWS/σ	[60,120]	0.62+0.02	0.85
	dried	α / S_{OH}	[-4,46]	0.36±0.04	1
		S_{TMPS}	[10,58]	0.51±0.09	1
		MWS	[70,120]	0.61±0.05	0.93±0.08
		σ	[70,120]	1	1

* data fitted in the isochronal representation of M´´ with Gaussian functions

S6. Fenofibrate incorporated in functionalized mesoporous silica nanoparticles (FNB@MSN_APTES and FNB@MSN_TMPS)

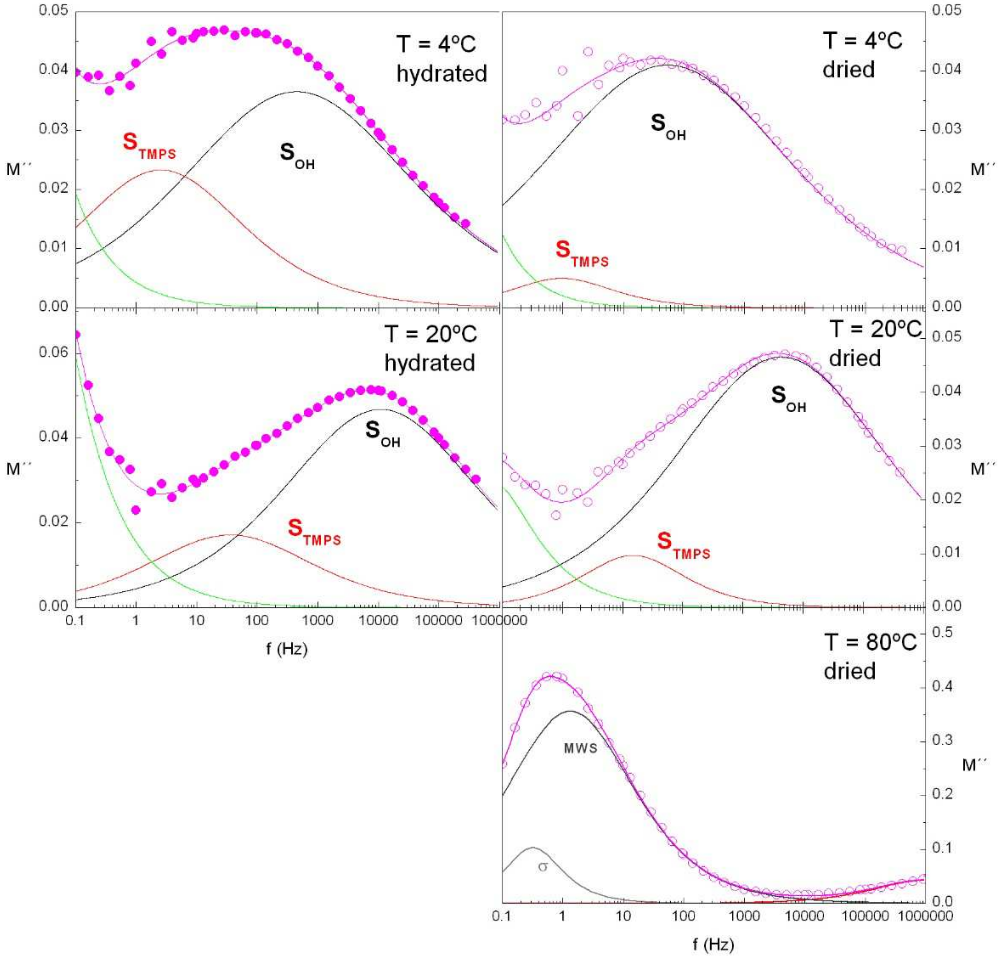

Figure S17. Representative isothermal spectra M´´ *vs*. f of the sample FNB@MSN_TMPS, pink circles; dotted lines represent the HN individual fitting function (imaginary part), and pink line is the overall fitting curve.

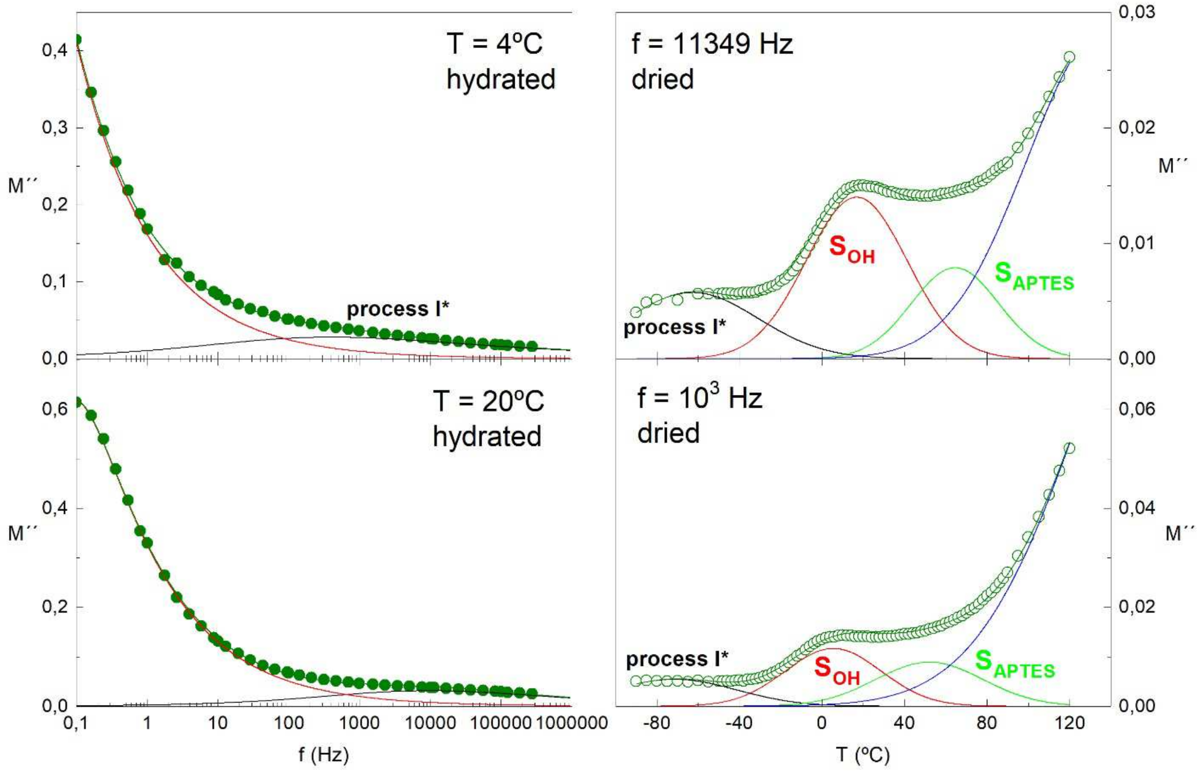

Figure S18. Representative isothermal spectra M´´ *vs.* f of the sample FNB@MSN_APTES, green circles; (a) and (b) thin lines represent the HN individual fitting function (imaginary part), and green line is the overall fitting curve. (c) and (d) isochronal representation (M´´ *vs.* f) of isothermal data taken on dehydrated sample; thin lines correspond to gaussians fitting functions and green line is the overall fitting curve.

S7. Transport properties

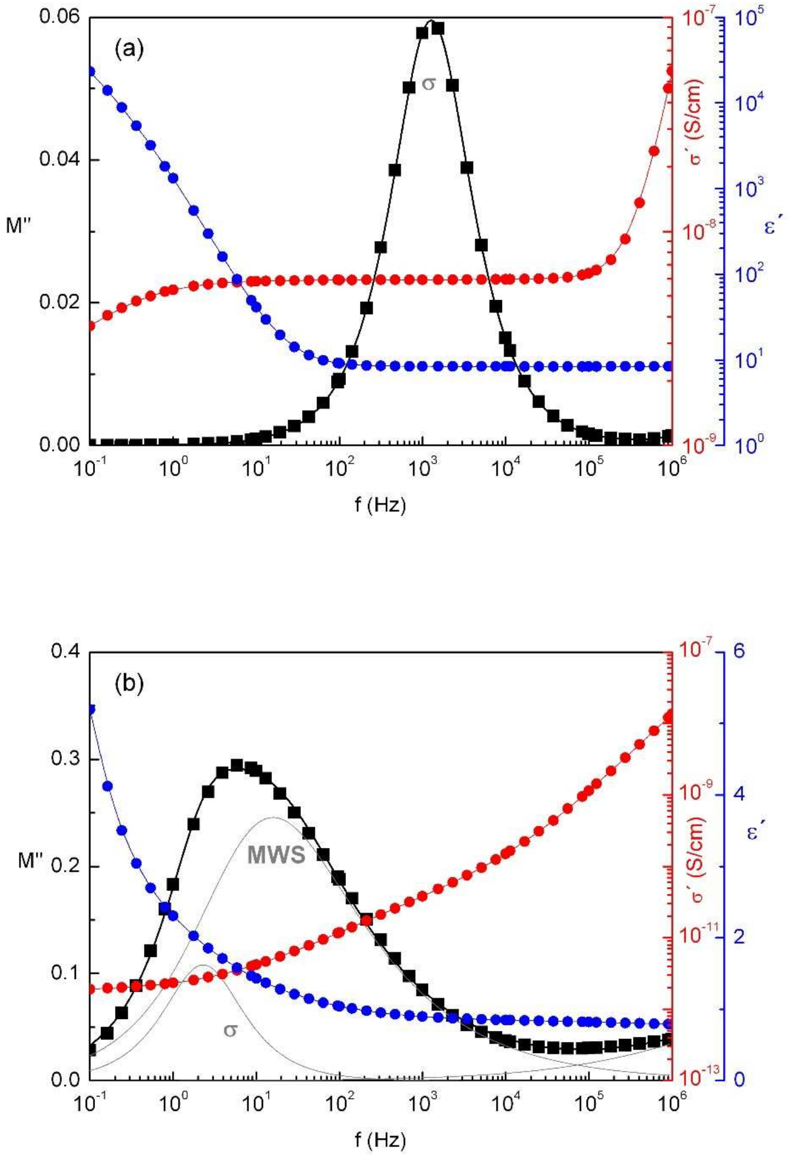

Figure S19. Isothermal spectra collected at 84 °C for (a) FNB and (b) FNB@MSN: M´´ as black squares in left axis, σ´ as red circles in right axis, and ε´ as blue triangles in secondary right axis.

In bulk FNB, the dc conductivity involves a large frequency range to which corresponds a symmetric peak in M´´ and a plateau in ε´. When FNB is incorporated in MSN, the σ′ spectrum exhibits a frequency dependence behavior possibly related to the presence of another relaxation process (MWS) besides that related to dc conductivity.

S8. References

[1] H. P. Diogo, M. T. Viciosa, J. J. Moura Ramos. Differential scanning calorimetry and thermally stimulated depolarization currents study on the molecular dynamics in amorphous fenofibrate. *Thermochimica Acta* **2016**, 623, 29–35.

[2] U. Sailaja, M. S. Thayyil, N. S. K. Kumar, G. Govindaraj. Molecular dynamics of amorphous pharmaceutical fenofibrate studied by broadband dielectric spectroscopy. *J. Pharmaceutical Analysis* **2016**, 6, 165–170.

[3] R. Böhmer, K. L. Ngai, C. A. Angell, D. J. Plazek. Nonexponential relaxations in strong and fragile glass formers. *J. Chem. Phys.* **1993**, 99, 4201–4209.

[4] G. P. Johari, M. Goldstein. Viscous liquids and the glass transition. II. Secondary relaxations in glasses of rigid molecules. *J. Chem. Phys.* **1970**, 53, 2372–2388.

[5] K. L. Ngai, E. Kamińska, M. Sekuła, M. Paluch. Primary and secondary relaxations in bis-5-hydroxypentylphthalate revisited. *J. Chem. Phys.* **2005**, 123, 204507.

[6] K. L. Ngai. An extended coupling model description of the evolution of dynamics with time in supercooled liquids and ionic conductors. *J. Phys.: Condens. Matter* **2003**, 15, S1107–S1125.

[7] K. L. Ngai, M. Paluch. Classification of secondary relaxation in glass-formers based on dynamic properties. *J. Chem. Phys.* **2004**, 120, 857–873.

[8] F. Álvarez, A. Alegría, J. Colmenero. Relationship between the time-domain Kohlrausch-Williams-Watts and frequency-domain Havriliak-Negami relaxation functions. *Phys. Rev. B* **1991**, 44, 7306–7312.

[9] A. Afzal, M. S. Thayyil, P.A. Sivaramakrishnan, S. Urpayilc, S. Capaccioli. Molecular dynamics in the supercooled liquid and glassy states of bezafibrate and binary mixture of fenofibrate. *J. Non-Crystal. Solids* **2020**, 550, 120407 (1–13).

[10] G. Szklarz, K. Adrjanowicz, M. Dulski, J. Knapik, M. Paluch. Dielectric relaxation study at ambient and elevated pressure of the modeled lipophilic drug fenofibrate. *J. Phys. Chem. B* **2016**, 120, 11298−11306.
